# Analysis of the Current State of Research on Bio-Healing Concrete (Bioconcrete)

**DOI:** 10.3390/ma17184508

**Published:** 2024-09-13

**Authors:** Alexey N. Beskopylny, Evgenii M. Shcherban’, Sergey A. Stel’makh, Alexandr A. Shilov, Andrei Chernil’nik, Diana El’shaeva, Vladimir A. Chistyakov

**Affiliations:** 1 Department of Transport Systems, Faculty of Roads and Transport Systems, Don State Technical University, 344003 Rostov-on-Don, Russia; 2 Department of Engineering Geometry and Computer Graphics, Don State Technical University, 344003 Rostov-on-Don, Russia; au-geen@mail.ru; 3 Department of Unique Buildings and Constructions Engineering, Don State Technical University, Gagarin Sq. 1, 344003 Rostov-on-Don, Russia; sergej.stelmax@mail.ru (S.A.S.); alexandr_shilov@inbox.ru (A.A.S.); chernila_a@mail.ru (A.C.); diana.elshaeva@yandex.ru (D.E.); 4 Center for Agrobiotechnology, Don State Technical University, Gagarin Sq. 1, 344003 Rostov-on-Don, Russia; vladimirchi@yandex.ru; 5 Laboratory of Mechanics of Multicomponent and Multiphase Media, Peter the Great St. Petersburg Polytechnic University (SPbPU), 195251 St. Peterburg, Russia; 6 D.I. Ivanovsky Academy of Biology and Biotechnology, Southern Federal University, Stachky 194/1, 344090 Rostov-on-Don, Russia

**Keywords:** bacteria, bioconcrete, crack healing, durability, self-healing concrete, compressive strength

## Abstract

The relatively small tensile strength of concrete makes this material particularly vulnerable to cracking. However, the reality is that it is not always possible and practically useful to conduct studies on high-quality sealing cracks due to their inaccessibility or small opening width. Despite the fact that currently there are many technologies for creating self-healing cement composites, one of the most popular is the technology for creating a biologically active self-healing mechanism for concrete. It is based on the process of carbonate ion production by cellular respiration or urease enzymes by bacteria, which results in the precipitation of calcium carbonate in concrete. This technology is environmentally friendly and promising from a scientific and practical point of view. This research focuses on the technology of creating autonomous self-healing concrete using a biological crack-healing mechanism. The research methodology consisted of four main stages, including an analysis of the already conducted global studies, ecological and economic analysis, the prospects and advantages of further studies, as well as a discussion and the conclusions. A total of 257 works from about 10 global databases were analyzed. An overview of the physical, mechanical and operational properties of bioconcrete and their changes is presented, depending on the type of active bacteria and the method of their introduction into the concrete mixture. An analysis of the influence of the automatic addition of various types of bacteria on various properties of self-healing bioconcrete is carried out, and an assessment of the influence of the method of adding bacteria to concrete on the process of crack healing is also given. A comparative analysis of various techniques for creating self-healing bioconcrete was performed from the point of view of technical progress, scientific potential, the methods of application of this technology, and their resulting advantages, considered as the factor impacting on strength and life cycle. The main conditions for a quantitative assessment of the sustainability and the possibility of the industrial implementation of the technology of self-healing bioconcrete are identified and presented. Various techniques aimed at improving the recovery process of such materials are considered. An assessment of the influence of the strength of cement mortar after adding bacteria to it is also given. Images obtained using electron microscopy methods are analyzed in relation to the life cycle of bacteria in mineral deposits of microbiological origin. Current gaps and future research prospects are discussed.

## 1. Introduction

Modern construction cannot be imagined without concrete, which is one of the main structural materials in this industry. Its widespread use is due to its versatility and accessibility, as well as outstanding physical and mechanical properties [1,2,3]. The use of concrete is not limited to building structures and goes far beyond the construction of urban infrastructure. Of great interest is the use of concrete in structures that have special requirements, such as structures in contact with bodies of water. Concrete in its various forms has been present since ancient times [4,5]. Modern concrete is made by mixing ordinary Portland cement, coarse and fine aggregates, and water in the required proportions. Often, additional modifying components are added to the concrete mixture, which improves the properties of both the fresh mixture and the final material [6,7,8,9,10]. However, an inherent disadvantage of concrete is cracks, which inevitably appear at the stage of hardening and accompanying shrinkage of this material, regardless of the composition and ratio of the components of the concrete mixture. Cracks that occur during shrinkage and the further operation of the element over time lead to the degradation of the material due to the ingress of moist air, water, and aggressive chemicals, such as acid gases, various chemicals, and salts, which, in conditions of high humidity and large amounts of atmospheric precipitation, can disrupt the microstructure of concrete and ultimately lead to the destruction of the element. Cracks also provide access to the steel reinforcement component of reinforced concrete structures, thereby making it vulnerable to corrosion and reducing the service life of the reinforced concrete element [11].

The reduced durability of concrete due to cracks has been a pressing problem throughout the history of this material. Technologies that prevent the occurrence of large cracks, as a rule, allow for the occurrence and development of micro-cracks, which, despite the small opening width, also lead to material degradation. Currently, in mass construction, the problem of the occurrence of cracks does not have a solution that is relevant for widespread use, which means a different approach is needed. The very idea of the autonomous healing of cracks, by analogy with the regeneration of living organisms, has long been relevant and attracted close attention from researchers, but the possibility of its implementation has appeared only in recent decades.

**Figure 1:**
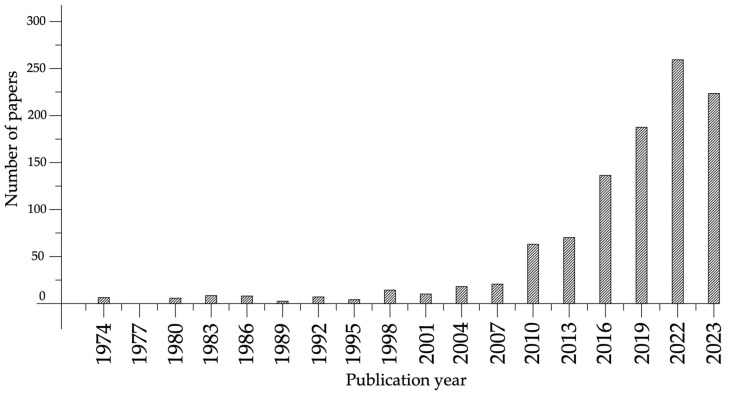
Research on self-healing concrete.

Today, there are several approaches to creating self-healing materials. Primarily, this involves a restoration using chemical agents located inside brittle fibers or microcapsules, which, when damaged during crack development, release an air-hardening agent that fills the crack. This method is notable in that it does not impose restrictions on the chemical agents used, allowing the use of new compositions for filling cracks. The disadvantages of this method include the fact that it is suitable for filling relatively small cracks, and that the amount of filling agent is limited by the number of microcapsules or fibers damaged by the crack, which negatively affects the restoration process of the material. Also important is the question of the durability of chemical agents for filling cracks and their relevance during the service life of the concrete element, which can significantly exceed the shelf life of the restoring composition. Moreover, the earlier a crack appears in concrete, the more likely it is that it will develop faster than cracks that appear after a long period of concrete operation. Therefore, for cracks that appear at different stages of the material’s life cycle, various self-healing processes can be applied, depending on the method of self-healing, the mechanism of the process, and the parameters of the cracks and the speed of their development. Figure 1 shows the number of studies on self-healing concrete over the past few decades. A sharp increase in the popularity of this topic over the past 10 years is clearly noticeable.

As part of the current study, Web of Science, ScienceDirect, ResearchGate, SpringerLink, MDPI, Taylor and Francis, and other databases were used to search for materials. The keywords used were “bacterial”, “self-healing”, “calcium carbonate crystals” and others.

A separate group can be considered for a method in which modifiers are added to the concrete mixture, which are activated using special conditions, for example, high temperature. The clear advantage of this technology is its ease of use and the possibility of mass use, as well as its relevance throughout the entire service life of the structure; however, it also has a significant disadvantage, which is the very narrow scope of its application, limited by the properties of materials that can withstand the conditions of activation of filling agents, for example, high temperature.

There is an extremely remarkable technology for creating self-healing concrete using an agent of natural biological origin that can eliminate cracks, crevices and open pores in concrete. It consists of adding bacteria to the concrete mixture, which, in unfavorable living conditions, form resistant spores that can remain potentially active for many decades. The restorative factor of this biological agent is the formation of a large number of calcium carbonate crystals during the life of the bacteria, which fill cracks. The advantages of this technology are obvious: it does not require a lot of time and money or a large industrial infrastructure for its implementation. This technology is completely autonomous and can actively work even in structures that cannot be supervised.

Given that concrete with a biological self-healing agent is able to successfully and stably heal cracks in concrete with minimal input of resources, such a concept has become a very attractive area for researchers interested in finding a sustainable and cost-effective method for creating self-healing cementitious composites that would not require manual interventions. To effectively solve this problem, one can resort to implementing an autonomous crack-healing process using natural processes by adding a certain type of bacteria to concrete when mixing its components. Even the basic process of bacterial cellular respiration produces enough carbon dioxide to regenerate concrete cracks. The process of decomposition of urea is more effective. Some microorganisms, for example *Bacillus subtilis* and *Bacillus Pasteurii*, can cause an enzyme known as urease to bind to a calcium-based nutrient medium, resulting in the formation of calcium carbonate crystals that seal micro-cracks in concrete. This process of sealing micro-cracks through biomineralization is a promising technology for creating self-healing cementitious composites and can also significantly improve the strength properties of concrete and its durability. Also, optimizing the concentration of bacteria can improve results by filling open pores in concrete with calcium carbonate crystals [12]. Recently, there have been reports that not only bacteria, but mold fungi that produce urease can be used to heal micro-cracks of concrete [13].

An analysis of the existing sources shows that the encapsulation method, in which bacteria and a nutrient medium are introduced into a concrete mixture in “special microcapsules that release the contents when damaged during cracking”, is more effective in terms of the strength and durability of concrete than the method in which bacteria and a nutrient medium are added directly to the concrete mixture [14]. The effectiveness of autonomous bacterial self-healing of concrete when using the encapsulation method directly depends on the type of microcapsules and their content, the resistance of the capsule to mechanical stress during mixing and laying the concrete mixture, the uniformity of the distribution of microcapsules in the concrete matrix and the possibility of its control, as well as on the degree of restoration of the mechanical and strength characteristics of a material. Despite the fact that the restoration of cracks with an opening width of more than 0.8 mm is a difficult task, healing them with a biological agent by filling them with calcium carbonate crystals is possible [15].

The use of self-healing technology based on biological agents is especially effective in lightweight concrete that uses lightweight coarse aggregate, as it creates a more favorable environment for bacteria, which leads to a significant increase in recovery ability, structural stability and durability [16]. The strength of concrete increases due to the formation of calcium carbonate crystals in concrete at any age when rice husk ash additive is included in concrete [17]. The use of biological crack-healing technology can also increase the strength of structural concrete of the highest grade 50 MPa with the highest calcium carbonate content by 24% [18]. A reduction in concrete porosity and water absorption was also noted, resulting in a 22% increase in strength compared to conventional concrete due to the inclusion of the bacteria *Sporosarcina pasteurii* in the mixture [18]. A similar method of improving the process of calcium carbonate formation, but with the help of different types of fungi, was used in studies [19,20,21,22,23].

The technology for creating self-healing cement composites based on a biologically reducing agent involves adding not only bacteria to the mixture but also various types of nutrient media rich in calcium. Typically, autonomous recovery in these cases is achieved through the growth of calcium carbonate crystals due to the direct mineralization of calcium compounds by bacterial species such as *Bacillus subtilis* [24], or due to the decomposition of urea by ureolytic bacteria such as *Bacillus sphaericus* [25]. Calcium carbonate crystals produced by bacteria combine well with concrete and are also safe for the environment [26]. Autonomous self-healing due to the activity of microorganisms is a relatively new concept that has become possible due to advances in biotechnology that have occurred quite recently. This technology is not only environmentally friendly, cost-effective compared to analogs and easily applicable to mass production, but also safe, since *Bacillus sphaericus* bacteria are safe for humans [27].

To the above, it is also necessary to add that the vital activity of bacteria consumes oxygen, which significantly reduces its content in the concrete matrix, which additionally protects steel reinforcement from corrosion. Also, calcium carbonate crystals produced by microorganisms seal pores and micro-cracks, preventing the access of water, moist air and aggressive substances into the concrete body, and also additionally bind grains of fine and coarse aggregate, increasing the strength and durability of the material. At the same time, energy consumption and greenhouse gas emissions during the production of bacteria, nutrient medium and their growth are insignificant. The bacteria of the genus *Bacillus* are characterized by the fact that in conditions of a highly alkaline environment and high humidity, they form spores that are resistant to unfavorable and aggressive environments, which allow them to survive in unfavorable conditions for hundreds of years. This ability allows them to be used as an autonomous self-healing agent that does not require special conditions and does not have a shelf life shorter than the service life of the concrete element. Thus, the bacteria of the genus *Bacillus* are the main favorite for use as biologically active self-healing agents [28,29,30,31]. The self-healing properties of biologically active reducing agents are usually assessed by adding bacteria and a nutrient medium directly to the concrete mixture, or by encapsulation, that is, by adding microcapsules containing bacteria and a nutrient medium to concrete [32].

A thorough examination of the constituent elements of self-healing bioconcrete is provided in this review, along with a meticulous assessment of the diverse formulation techniques employed in its production, while considering specific input and output variables. Additionally, consideration is given to recent publications exploring the physical, mechanical, and microstructural properties of self-healing bioconcrete, and their resilience over time. A prime focus in this study regards the key factors that influence the properties of self-healing bioconcrete from the initial stage to full hardening.

Therefore, the objective of this study is to establish an empirical and literary foundation for future research and the focused enhancement of the global theory of self-healing bioconcrete. The study aims to conduct a comprehensive literature review and an analysis of the current state of the self-healing bioconcrete market in global construction and production. It also seeks to assess prospects and identify specific vectors for its development. In order to accomplish the objective, it is imperative to address the subsequent tasks:

(1) Conducting a thorough review of the latest global investigations, taking into account the best existing global practices and scientific achievements in the self-healing bioconcrete sphere;

(2) The determination of the constituent, technologically and structurally effective, scientific approaches to consider the problems of achieving the environmental and economic efficiency of self-healing bioconcrete for a wide variety of types of countries, regions, climatic zones, buildings and structures of different levels of durability;

(3) The identification of the primary factors and criteria determining the performance and quality of self-healing bioconcrete;

(4) The determination of the fundamental correlations between the composition components, microstructure and performance of self-healing bioconcrete;

(5) Identifying the relationship at the micro- and macro-levels in the formation of the structure and properties of self-healing bioconcrete;

(6) An assessment of the raw material elements, the right proportion of the composition; identifying the relationship of these compositions and proportions on the performance of self-healing bioconcrete and influence on the building’s durability; a quantitative and qualitative assessment of these relationships between the components of self-healing bioconcrete, performance and power consumption.

The article has the following structure: Introduction (Section 1); Methods (Section 2); the results, in the form of sections detailing the basic principles of the technology for the autonomous restoration of concrete using bacteria (Section 3), the process of introducing a bacterial self-healing agent into a concrete mixture (Section 4), the process of the autonomous restoration of concrete using biomineralization (Section 5), the effect of an autonomous bacterial-based reducing agent on the properties of concrete (Section 6), the influence of additives in the form of a nutrient medium for bacteria and microcapsules on the physical and mechanical properties of concrete (Section 7), the physical and mechanical properties of self-healing concrete restored using a bacterial reducing factor (Section 8), the features of the macro-, micro- and nanostructure of self-healing bioconcrete (Section 9), impact on the environment (Section 10); advantages and prospects for further research on self-healing bioconcrete (Section 11), and the Discussion and Conclusions (Section 12).

## 2. Methods

The following databases were used for the review: Web of Science, ScienceDirect, ResearchGate, SpringerLink, MDPI, Taylor and Francis and others (about 10). The key words were “bacterial”, “self-healing”, “calcium carbonate crystals”, “self-healing concrete”, “concrete durability” and others.

**Figure 2:**
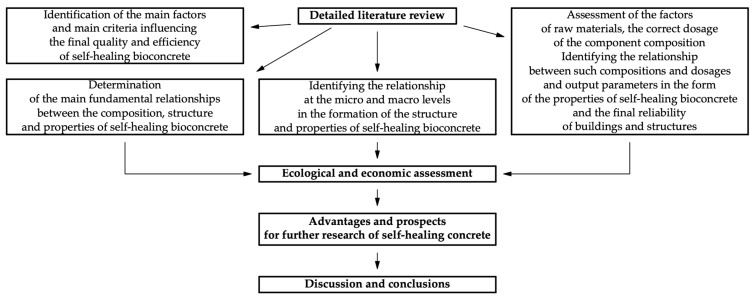
Flow chart of the research methodology.

The study was carried out using methods such as a review of world-class research on the topic of self-healing bioconcrete using the databases of international journals, the study of patented technologies, and the study of the raw material base in relation to each region of the world. The selection of source material for the review was carried out, taking into account the journal, the year of publication, the compliance of the material with keywords, and the topic of this review article. The initial data were information about the composition, formulation, technology, and properties of various self-healing bioconcrete composites created and used in various regions of the world. A significant amount of research data on the properties and structural characteristics of self-healing bioconcrete have been identified. The parameters of self-healing bioconcrete most often considered in scientific articles are the rate of crack healing, the maximum opening width of a healed crack, compressive strength, tensile strength, bending tensile strength, elastic modulus, hardening time, workability, shrinkage, and structural changes occurring during the process of hardening of the material. The fundamental interactions occurring at micro- and macro-levels in self-healing bioconcrete were studied. To take into account and analyze risks when conducting research, a subject area was identified from those literary sources that have similar features to each other, thereby making it possible to achieve some verification of the results of some authors based on the results of other authors. Visualization methods and tabular methods were used. The research methodology is presented in the form of a block diagram in Figure 2.

## 3. Basic Principles of the Technology for the Autonomous Restoration of Concrete Using Bacteria

The practice of autonomous healing of cracks in concrete using a bioactive agent is an effective approach to repair micro-cracks in concrete and shows good results for concrete elements. Ideally, this technology should recognize various types of damage to concrete, damage to its integrity, and the occurrence of cracks that would lead to the release of bacteria. During their life, bacteria form a layer of calcium carbonate crystals on the concrete surface inside the cracks. The bacteria introduced within the framework of this technology survive well in the alkaline environment of the concrete matrix [33,34]. In such an environment, the bacteria *Bacillus sphaericus* convert urea into ammonium and carbon dioxide, which interacts with calcium hydroxide. As a result, calcium carbonate CaCO_3_ is formed [35], precipitating in form of crystals. Thus, calcium carbonate formed as a product of the vital activity of bacteria seals micro-cracks and pores, and also improves the adhesion of cement paste to coarse and fine aggregates, thereby increasing its physical and mechanical characteristics [36]. The results of the study [37] showed that cracks with an opening width of up to 0.2 mm are quickly healed by concrete independently, while cracks with an opening width of more than this value could not recover independently. When micro-cracks occur in concrete containing a biologically active reducing agent, air and moisture enter it, which create conditions for bacteria to emerge from hibernation, that is, from the state of “dormant” inactive spores resistant to aggressive influences, into a state of active life, after which the autonomous recovery phase begins. The authors of [38] found that the formation of calcium carbonate crystals is the main factor in promoting crack healing. As soon as the micro-cracks are filled with calcium carbonate crystals, their access to air and moisture stops and the bacteria form spores, returning to a dormant state. The occurrence of other subsequent cracks will also cause bacteria to become active and seal new cracks. This mechanism uses the natural process of biomineralization in such a way that the bacteria act as a constant reducing agent. However, some types of bacteria can produce calcium carbonate crystals indirectly depending on different metabolic pathways. The different types of bacteria used in scientific research on the self-healing of concrete are presented in previous research. The bacteria of the genus *Bacillus* are most often used in research on self-healing building materials [39,40]. We can specify such species as follows: *B. cereus* [41], *B. pasteurii* [42,43,44], *B. megaterium* [45,46], B. *subtilis* [47], *B. sphaericus* [48,49,50,51,52,53], *B. pseudofirmus* [54,55] *B. alkalinitrilicus* [56]. However, the list of species relevant to this topic is not limited to bacilli. *Knoellia flava* [57], *Halomonas* sp. [58,59,60], *Micrococcus* sp. [61], and *Myxococcus xanthus* [62] were applied, as well as complex mixtures with unidentified components (microbiota inhabiting stone [63], *Mytilus californianus* shell extracts [64,65], etc.)

In heterotrophic processes, the formation of calcium carbonate crystals occurs more abundantly than in autotrophic ones. The cell walls of bacteria used to create self-healing concrete are coated with a thin layer of calcium carbonate [66,67]. For technical purposes, the most popular mechanism for researchers to decompose urea using bacteria is the use of the enzyme urease. This enzyme, produced by ureolytic bacteria, causes urea carbonate and ammonium to catalyze in the bacterial environment, causing an increase in pH and carbonate concentration. In [53], the authors suggested that the process of formation of calcium carbonate crystals is influenced by such factors as the concentration of calcium ions, the pH of the solution, the concentration of inorganically dissolved carbon, and the accessibility of nucleation sites. The first three factors are determined by the concrete matrix, while the last factor depends on the bacterial cell wall. The formation of calcium carbonate crystals by bacteria can be obtained in various ways, such as the transformation of calcium compounds such as calcium lactate or the hydrolysis of urea during bacterial activity. The first mechanism involves cracks allowing oxygen to enter the concrete, causing bacteria to convert calcium lactate into calcium carbonate at the crack site. A similar reaction of portlandite particles with CO_2_ produces more calcium carbonate than is needed to heal cracks. Thus, this mechanism may be useful for fresh concrete, in which anhydrate calcium hydroxide particles are still present [68].

The process of ureolysis or the decomposition of urea occurs thanks to the enzyme produced by ureolytic bacteria—urease, which catalyzes the hydrolysis of urea to ammonium and carbonate [69]. As a result, 1 mole of urea is hydrolyzed into 1 intracellular mole of ammonia and 1 mole of carbamic acid [35]. In the presence of microbial urease, the reaction proceeds as follows:

(1) $$\mathrm{CO}{\left({\mathrm{NH}}_{2}\right)}_{2}+H_{2}O-\mathrm{Hydrolysis}\to {\mathrm{NH}}_{2}{\mathrm{COOH}+\mathrm{NH}}_{3}$$

The ammonia and carbamic acid formed as a result of reaction (1) are then impulsively hydrolyzed into 1 mol of ammonia and 1 mol of carbonic acid, as shown in reaction (2) [69].

(2) $${\mathrm{NH}}_{2}{\mathrm{COOH}+H}_{2}O\to {\mathrm{NH}}_{3}+H_{2}{\mathrm{CO}}_{3}$$

Bicarbonates and 2 moles of ammonia and hydroxide ions with water are generated by ammonia and carbonic acid, as seen in reactions (3) and (4). Reactions (3) and (4) are reversible.

(3) $$2{\mathrm{NH}}_{3}+{2H}_{2}O\to {2\mathrm{NH}}_{4}^{+}+2{\mathrm{OH}}^{-}$$

(4) $$H_{2}{\mathrm{CO}}_{3}\to {\mathrm{HCO}}_{3}^{-}+H^{+}$$

The formation of hydroxide ions from the reaction increases the pH level so that the bicarbonate balance can be altered to produce carbonate ions (5).

(5) $${\mathrm{HCO}}_{3}^{-}+H^{+}+2{\mathrm{NH}}_{4}^{+}+2{\mathrm{OH}}^{-}↔{\mathrm{CO}}_{3}^{-2}+2{\mathrm{NH}}_{4}^{+}+2H_{2}O$$

When calcium ions are present in the form of calcium carbonate crystals, they precipitate (6).

(6) $${\mathrm{Ca}}^{+2}+{\mathrm{CO}}_{3}^{-2}↔{\mathrm{CaCO}}_{3}$$

During this process, a monolayer of calcite is formed, and subsequently several such layers are formed. The cell wall of bacteria has a negative charge. Bacteria receive cations from the atmosphere along with Ca^2+^ ions, which are deposited on the cell surface. Ca^2+^ ions react with CO^2^ to form calcium carbonate crystals on the cell surface in the nucleation region.

(7) $${\mathrm{Ca}}^{2+}+\mathrm{Cell}\to \mathrm{Cell}-{\mathrm{Ca}}^{2+}$$

(8) $$\mathrm{Cell}-{\mathrm{Ca}}^{2+}+{\mathrm{CO}}_{3}^{2-}\to \mathrm{Cell}-{\mathrm{CaCO}}_{3}$$

Many microorganisms can manifest the urea decomposition mechanism to precipitate calcium carbonate.

Thus, autonomous crack healing with a bacterial repair factor can be achieved through any of these mechanisms. However, a number of factors, namely access to water, the opening width and surface area of the crack to be healed, healing time, and bacterial lifespan, are also very important for the effective implementation of self-healing technology based on biological healing agent.

A comprehensive study of the research sources has uncovered particular utilizations of bacteria. *Bacillus aerius* bacteria introduced into concrete along with rice husk ash increased the durability of concrete [17]. The bacteria Bacillus megaterium added to cement demonstrated a rise in the concrete compressive strength by 24% [17]. *Bacillus sphaericus* improved the strength of concrete through significant amounts of calcium carbonate deposition [18,70]. *Sporosarcina pasteurii*, when applied to the in-concrete mixture with the addition of fly ash, also improved its strength [71]. When incorporated into concrete with the addition of silica fume, *Sporosarcina pasteurii* also showed an increase in the strength and durability of the material [19]. The introduction of *Bacillus sphaericus* bacteria into concrete has also been studied to create an autonomous treatment of the concrete surface. The results showed that concrete with the addition of these bacteria does not require additional surface treatment [49].

## 4. The Process of Introducing a Bacterial Self-Healing Agent into a Concrete Mixture

In the literature, there are two main techniques for introducing a biological self-healing agent into concrete. As a rule, this is the addition of bacteria and a nutrient medium to the mixture directly when mixing the concrete components, or the addition of microcapsules containing bacteria and a nutrient medium to the concrete mixture. The first method involves adding bacteria to concrete along with graphite nanoplatelets, which are a suitable carrier for bacteria. Studies have shown that when adding bacteria directly to a concrete mixture, from the point of view of increasing the strength of concrete, the optimal amount is 30 × 10^5^ CFU/mL (Colony-Forming Units, an indicator of the number of viable microorganisms in a unit volume in a liquid (mL)) [17]. In a study [49], when *Shewanella* sp. bacteria were introduced directly into concrete, the compressive strength after 28 days of hardening was 25% higher compared to the control composition. In relation to the method of encapsulating bacteria in concrete, the method of impregnation and subsequent encapsulation of lightweight aggregate granules into a layer of polymer coating has shown effectiveness in terms of improving the overall characteristics of self-healing concrete [72].

**Figure 3:**
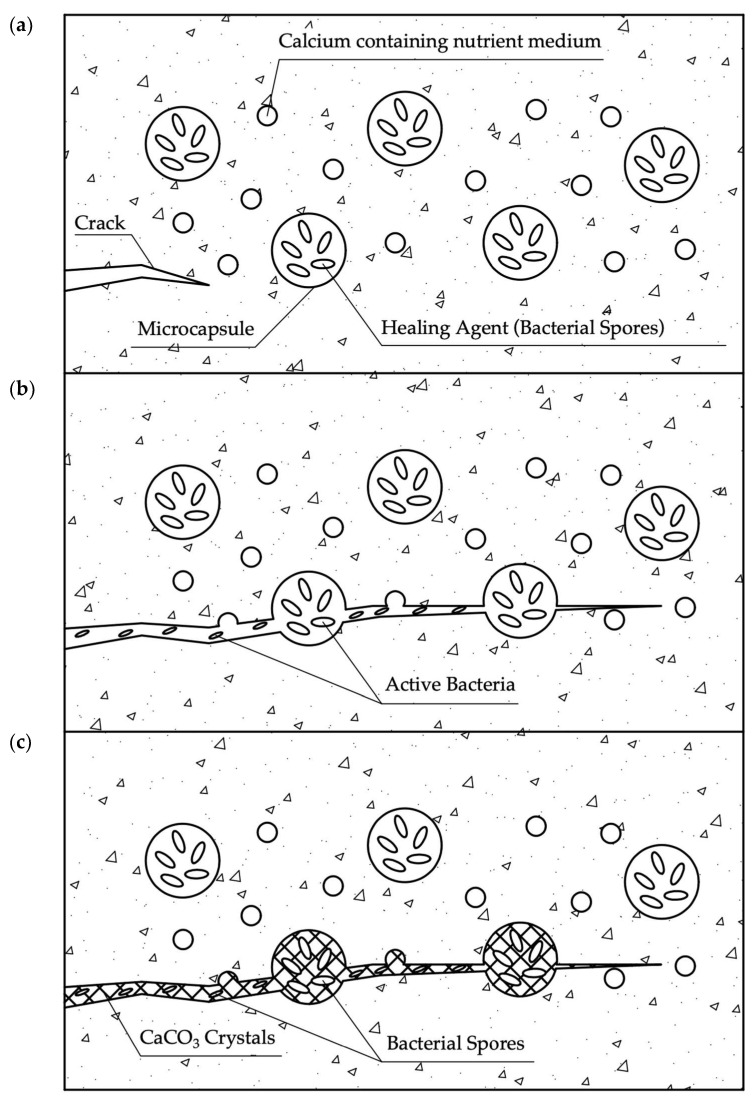
Activation process during crack formation of a biological self-healing agent added to concrete using microcapsules: (**a**) concrete mixture with embedded self-healing bacterial agent; (**b**) releasing a self-healing bacterial agent; (**c**) sealing the crack with calcium carbonate crystals.

The process of bacteria release from microcapsules is shown in Figure 3.

A self-healing bacterial agent embedded in the concrete mixture in microcapsules that are evenly distributed in the concrete matrix is released when the microcapsule is damaged by a crack. The healing agent, by capillary movement, comes into contact with the surfaces of the crack and connects with the nutrient medium, also dispersedly distributed in the body of the concrete element, which begins the process of formation of calcium carbonate crystals, which seal the crack to the entire depth of its penetration. According to the study [72], it was found that microcapsules in cement composites are successfully preserved during the mixing of the concrete mixture and during its hardening, and also act effectively in the event of cracking.

The method of encapsulating bacteria in a concrete mixture with a sufficient volume of microcapsules can show impressive results in crack healing [73,74]. Bacterial spores introduced in concrete in hydrogel microcapsules improved the efficiency of the restorative effect, both in terms of the volume of calcium carbonate crystals and in terms of the rate of crack healing [71]. Overall, the encapsulation method showed good results due to a more uniform distribution of microcapsules, the additional protection of microorganisms from the highly alkaline environment of fresh concrete, and the effectiveness of autonomous recovery in terms of the rate and volume of calcium carbonate crystal formation.

In [75], the authors investigated the relevance of using the spores of three species of bacteria, *Bacillus cohnii*, *Bacillus halodurans* and *Bacillus pseudofirmus*, as a self-healing agent in concrete. Bacterial spores were added directly to the concrete, without the use of microcapsules. Yeast exhaust and peptone-based media were used to feed the bacteria. After curing, the concrete was tested for compressive and tensile strength. Significant negative variation was observed among bacterial and control samples. The examination of the samples using electron microscopy showed that after 12 days of incubation, the bacteria began to produce calcium carbonate crystals. The peculiarity of these studies is that they focused on the surface of concrete, where, under the conditions of this study, bacteria were fed in a special environment, after which the growth of calcium carbonate crystals was observed. In further studies, the authors [28] conducted experiments on the use of bacteria as a healing agent, which can remain viable in the body of concrete when added together with a nutrient medium in the form of yeast extract, peptone and a mineral precursor, which had previously shown a potential effect on the healing process. The authors studied two types of spores of alkaliphilic bacteria—*Bacillus pseudofirmus* and *Bacillus cohnii*. Calcium compounds—calcium acetate and calcium lactate—were chosen as mineral precursors. However, calcium lactate was the only compound in the study that improved the durability of concrete. The calcium carbonate crystals formed in this study ranged in size from 20 to 80 µm. At the same time, the authors found that the alkaline environment of concrete and a decrease in the size of micropores reduces the number of activated bacteria and, as a consequence, the formed calcium carbonate crystals. According to the authors, this mechanism has advantages over the enzymatic approach based on urease, due to the fact that such metabolic processes do not produce excess ammonia, which can have a destructive effect on the cement–concrete matrix and lead to increased corrosion [28].

The authors [15] studied the effect of crack opening width on healing conditions when introducing a healing agent in the form of spore-forming bacteria and a nutrient medium directly into concrete. The opening width of the cracks to be restored ranged from 0.1 mm to 0.5 mm. Healing was carried out at a constant temperature of +25 °C, with alternating humidity levels during hardening and with alternating wet and dry cycles. Crack healing was measured at concrete ages of 7, 14, 28, 60 and 90 days based on the area of healing for the specified period. When the samples were completely immersed in water, cracks with an opening width of up to 0.3 mm were completely sealed, while the degree of healing of cracks with an opening width from 0.1 mm to 0.3 mm was about 85%. The most effective methods for healing cracks were wet curing and alternating dry and wet cycles, while the rate of crack healing when alternating wet and dry cycles was lower compared to wet curing. The survival rate of spore-forming bacteria was much higher than that of other species used in the study. At the same time, a decrease in the porosity of concrete led to a reduction in the distance of the transportation of bacteria to the nutrient medium, which is why newly formed cracks healed more effectively than in later stages.

When it comes to autonomous self-healing technology for concrete, special attention is paid to the need for a well-organized crack-healing process that does not require additional intervention. In this case, the crack-sealing mechanism must be active and relevant throughout the entire service life of the concrete element. To ensure the above factors, the survival of the bacterial self-regenerating agent is very important. Despite much evidence from researchers about the effectiveness of the method of adding bacteria and a nutrient medium to the concrete mixture when mixing concrete components, a number of authors argue that this method has a number of additional nuances that put the survival of bacteria for a long time at risk. Thus, the author of the study [23] indicates that the period during which bacterial spores not protected by microcapsules are potentially active and ensure full operation of the self-healing mechanism of concrete is limited to only two months. As a result, active self-healing properties are observed in samples only at an early age. This can occur due to a number of reasons, including the highly alkaline environment of the cement matrix, mechanical stress during concrete mixing, and the process of cement hydration. The activity of bacterial spores can be significantly reduced due to prolonged exposure to a highly alkaline environment. In addition, some spores can be mechanically destroyed when mixing the components of the concrete mixture, either by the mixing mechanism or due to the collision of coarse aggregate grains. The hydration of cement over time leads to a decrease in the porosity of the material to a size of 0.5 μm, while bacterial cells used as a reducing agent in concrete, as a rule, are larger [24]. Consequently, the degree of activation of bacterial spores can be significantly reduced as a result of a decrease in pore size or their complete blockage at a later age of the material. To solve the problems described above, you can resort to encapsulating bacteria, which will protect them from the effects of the above aggressive factors of the concrete mixture and prevent the excessive and premature formation of calcium carbonate crystals. Bacteria can be encapsulated in a variety of ways, using materials such as expanded clay [23,76], silica gel, glass–polyurethane tubes, diatomaceous earth, hydrogel [77], and melamine-based microcapsules. It is completely predictable that the introduction of microcapsules into a concrete mixture reduces the performance characteristics of the prototype; however, at the same time, a self-healing biological agent introduced into the mixture by encapsulation also provides certain advantages, in the form of a reduction in the opening width and depth of cracks, and a reduction in water absorption and permeability, as well as the effective restoration of strength after healing [78]. The urea hydrolysis process is primarily used in the field to create self-healing bioconcrete, and in geotechnical and geological stabilization applications that include soil erosion control, underground crack healing, and for cementitious composites used in boreholes and wells [79]. The authors of [80] studied the healing coefficient of mortar samples placed in non-cohesive soil with changes in pH and soil moisture. For samples of mortar containing microcapsules with a biological self-healing agent, healing coefficient values in the range of 47–83% were obtained. Under the same conditions, a solution containing bacteria added without microcapsules showed a lower healing rate. The mechanism of bacterial encapsulation itself can be carried out using several methods: using polymer microcapsules, using lightweight aggregate, using special cement, and using special mineral compounds.

The authors of the study [25] used melamine-based polymer microcapsules to encapsulate *Bacillus sphaericus* bacterial spores into a concrete mixture. Calcium nitrate was used as a calcium-containing nutrient medium, which was added to the concrete along with other nutrients, namely urea and yeast extract. The healing of cracks was assessed by the ratio of the total area of healed cracks to the total area of cracks. Concrete with encapsulated spores showed a healing rate of 48% to 80%, while concrete without the encapsulation method showed only 50% total crack recovery. The greatest amount of healing was observed in samples that were subjected to alternating wet and dry cycles. The wet cycle lasted about 16 h. The longest length of the healed crack was approximately 970 mm. The content of microcapsules with a biological reducing agent equal to 5% of the volume gave more stable results, but significantly reduced the strength of concrete. The authors found that the optimal content of microcapsules is 3%.

One of the innovative ways to create microcapsules for the introduction of biologically active reducing agents into concrete is the use of hydrogel. A study [33] showed that the hydrogel makes it possible to provide the necessary moisture conditions for bacterial activity and the maximum activation of the process of autonomous restoration of concrete with minimal manual intervention. At the same time, the hydrogel also has sufficient properties to act as a mechanical protective shell for bacteria. The use of hydrogel in encapsulating bacteria in concrete showed healing rates of approximately 40% to 90% and also reduced the permeability of concrete by approximately 68%. The effectiveness of the autonomous healing of concrete using bacteria encapsulated in a hydrogel shell is due to the ability of the hydrogel to absorb and retain moisture. The authors of the study [31] found that the hydrogel is able to absorb and retain 70% of moisture during the first 12 h and 30% within 24 h after interaction with air with a relative humidity of 60% at a temperature of 20 °C. According to the authors, in regions with high air humidity and high rainfall, the use of hydrogels as a material for microcapsules in the production of self-healing bioconcrete may be the best option. Nonetheless, the properties of hydrogels may vary depending on their specific categorizations. The hydrogel can be ionic or non-ionic [24]. Ionic hydrogels are more responsive to the pH of the environment, resulting in their absorption capacity being highly dependent on the chemicals prevalent in the ambient air. Non-ionic hydrogels may be more suitable for self-healing bioconcrete applications because ionic moisture does not significantly affect their water absorption and retention capacity. Since studies regularly show an improvement in the process of autonomous restoration of bioconcrete when exposed to a humid environment, the use of hydrogels as a microcapsule material may be relevant for structures that do not have direct contact with water, since the hydrogel is able to actively and sufficiently absorb moisture from the air, which allows for the widespread use of such self-healing concrete in real structures. The authors of the study [79] analyzed the possibility of using a hydrogel based on sodium alginate as a material for microcapsules containing bacterial spores. The workability of the concrete mixture remained unaffected with the incorporation of 0.5% and 1% hydrogel by weight. At the same time, the hydrogel microcapsule, according to the authors, made it possible to preserve 99% of bacterial spores during the mixing of the concrete mixture. However, at 1% hydrogel microcapsule content by weight, a decrease in tensile strength of approximately 23.4% and compressive strength of approximately 30% was observed. This effect is presumably due to the formation of macrovoids caused by the addition of the hydrogel. That is, on the one hand, the hydrogel provides additional moisture to the concrete mixture during the hardening process, promoting strength gain, and on the other hand, it reduces the strength due to the formation of macropores. Despite the diametric opposite of these two effects, they can be balanced by varying the water–cement ratio and the dosage of hydrogel microcapsules. Thus, at a water–cement ratio above 0.45, the addition of hydrogel microcapsules has a much smaller effect on the process of concrete strength development. The authors of the study [81] used a water–cement ratio of 0.50, which is already a high enough value to reduce the strength of concrete containing hydrogel microcapsules.

**Figure 4:**
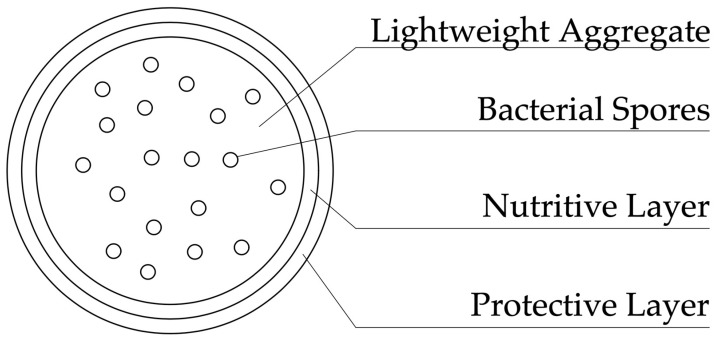
A diagram of the lightweight porous aggregate used in the technology for autonomously recovering bacterial concrete.

In a study [19], the authors used expanded clay as a precursor containing bacterial spores and calcium lactate. This approach is characterized by the fact that it does not use microcapsules, and the role of a protective shell is performed by a lightweight porous aggregate; when damaged, bacterial spores are activated and the process of autonomous restoration of concrete begins. The prototypes were immersed in water for two weeks, and the maximum opening width of the healed crack was 0.46 mm. The samples were then observed for 6 months. It was found that, within this period, the viability of bacteria did not decrease. Figure 4 shows a diagram of a lightweight porous aggregate used to encapsulate bacteria in concrete. Bacterial spores, in this case, are located in the porous structure of the filler, which is covered with a layer of nutrient medium and a protective layer.

Lightweight porous aggregates are usually used for the production of lightweight concrete, in which, in addition to the weight-reducing factor, they also act as an internal source of moisture, which is not only important for the concrete hardening process, but can also be useful for creating self-healing bioconcrete. However, the effectiveness of such aggregate is influenced by many factors, such as the amount of water in the aggregate, the grouping of aggregate grains in the concrete matrix, and the pore structure of the aggregate [82]. At the same time, lightweight porous aggregates significantly reduce the strength of concrete, which makes them unsuitable for use in heavy structural concrete, in which coarse aggregate makes up the majority of the concrete mass and has a decisive influence on its strength. In heavy concrete, as a rule, coarse aggregate is resistant to cracks, which, when formed, run along its surface. Moreover, in the case of lightweight porous aggregates, the crack will most likely pass through them, since the strength of such aggregates is most often much less than the strength of the cement gel. The authors of the study [24] observed a decrease in strength up to 50% after 28 days of concrete hardening, when using expanded clay instead of the classic coarse aggregate. Table 1 shows studies of lightweight self-healing bioconcrete with porous coarse aggregate with different types of biological healing agents.

The use of diatomite to introduce a biological reducing agent into concrete deserves special attention. Diatomite is found in abundance in silica and is the shell of microorganisms known as diatoms. They have an extremely porous structure and, as a result, can act as a capsule containing bacteria. In a study [29], the authors used diatomaceous earth to introduce spores of the bacteria *Bacillus sphaericus* into concrete. When a crack appeared and air and moisture entered it, the bacteria became active and began the process of the hydrolysis of urea and the production of calcium carbonate crystals from calcium nitrate used as a nutrient medium. The width of fully healed cracks ranged from 0.15 mm to 0.17 mm. At the same time, bacteria were predominantly concentrated in large pores of diatomite particles. According to the authors’ conclusions, the use of this method of introducing bacteria into concrete is an effective way to maintain the effective mineral-forming ability of bacteria in self-healing bioconcrete for a long time [99].

## 5. The Process of the Autonomous Restoration of Concrete Using Biomineralization

**Figure 5:**
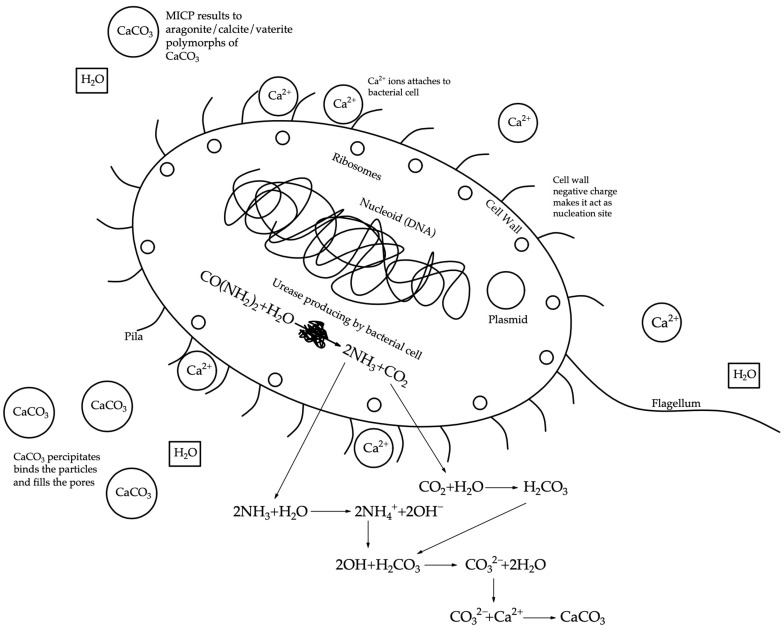
The process of calcium carbonate synthesis by a ureolytic bacterium using the example of *Sporosarcina pasteurii*.

The process of biomineralization is based on the production of minerals by living organisms. The mineralization of carbonic acid salts is often used in concrete technology, but in the case of biomineralization, the inorganic mineral phase is produced by living organisms [100]. Biomineralization can be biologically controlled and biologically induced. The first type is characterized by the formation and deposition of minerals in organic matrices or vesicles of microbial cells, which allows microorganisms to have significant control over the formation and development of minerals, as well as over their composition, deposit size, shape, and intracellular location. The second type is characterized by the release of metabolic products that react through ions with other components of the atmosphere, resulting in the deposition of mineral particles [101,102,103,104]. This biomineralization result is unintended and uncontrolled, resulting in selective cementation due to the formation of relatively insoluble organic and inorganic compounds. Such cementitious compounds are sometimes called biocement. Nowadays, it is a promising organic, environmentally friendly material associated with the process of the deposition of calcium carbonate on the walls of alkaliphilic bacteria. This process is widely used for heavy metal recovery [58,59,60], soil strengthening and improvement [105], stone element recovery [106,107], wastewater treatment [108], sand compaction [42], concrete strengthening [44], and increasing the durability of building materials [49,109]. The biomineralization process is shown in Figure 5.

Biocement consists of calcite formed during the metabolism of alkaliphilic bacteria and urea as a solution of calcium ions and substrate [110,111]. The enzyme urease is generated through the bacterial hydrolysis of urea, and calcium is utilized as a nutrient medium to facilitate biocement production. Biocement binds grains into a solid mass. Such a mechanism is applicable to soil stabilization [112,113], the construction of underground structures [114], the restoration of stone elements [115], the clogging of water channels, and the removal of pollutants [116]. For optimal adhesion, the even distribution of carbonates within the intergranular voids is imperative. The main feature of bacteria in the process of carbonate precipitation is their ability to create an alkaline environment, i.e., increase pH through physiological activity [117]. The increased alkalinity of materials such as lime and cement allows them to combine well with biocementation processes. Thus, the authors of the study [118] obtained biosandstone with a strength corresponding to natural sandstone by binding sandstone using high-alkaline biocementation. The *Shewanella* species, isolated from hot springs using biocementation, increased the compressive strength of cement mortar to 25% [42]. The authors of the study [92] used biocementation for the production of mortars, along with conventional cement. Bacteria selected by the authors were added to the water to combine the mixture of sand and cement. A cement ratio of 0.47 was selected for the 70.6 mm cubes. A similar composition was chosen for the manufacturing of concrete samples [96]. These samples showed increased compressive strength, by 17–36%, and a fourfold increased water resistance. Studies have shown that the biocementation process depends on the types of bacteria used, and that the effect of urease is very high, while the ability to precipitate calcium carbonate crystals can be called moderate. At the same time, it is the precipitation of calcium carbonate on the surface of the bacterial cell wall and in the concrete matrix that is the basis of biocementation. It is clear that large amounts of calcium carbonate crystal deposition are favored by a calcium-rich nutrient medium. Table 2 shows the healing capacity of some types of bacteria in self-healing concrete.

Traditional building materials such as brick, concrete, limestone or mortar are porous materials and can be protected by the biodeposition of bicarbonate compounds on their surface, sealing the open pores of such substrates [130]. This can be achieved by placing a colony of ureolytic bacteria on a porous surface by soaking or spraying. A number of researchers involved in the consolidation and strengthening of limestone have revealed the effectiveness of this technique by detecting the growth of calcium carbonate crystals on the surface of limestone caused by microbial activity [131]. To restore and protect limestone, the authors of the study [51] treated its surface with bacteria. In this study, 30 mm limestone cubes were soaked in a liquid bacterial medium containing 1% of a range of bacilli, which included five different types of *Bacillus sphaericus* and one type of *Bacillus lentus*, as well as NaHCO_3_, urea and CaCl_2_. Over the course of 4 weeks, several layers of CaCO_3_ were put on the surface of the limestone. The authors concluded that the bacteria with increased ureolytic ability reached the most visible and uniform layer of calcium carbonate crystal deposits.

The importance of bacterial biomineralization in natural rock restoration has been acknowledged for a considerable time [132]. Biocalcifying bacteria have been found to be suitable for the restoration of monuments or limestone parts of historical buildings [133]. A layer of calcium carbonate crystals on the surface of natural stone acts as a protective coating that closely follows its surface and does not hide its texture. However, a number of studies indicate that this method does not completely block the network of natural pores, which is why soluble salts can pass into the stone [102]. The destruction of concrete structures can occur for various reasons. These may include loads exceeding the design for this element, aggressive environmental conditions, and damage arising from unforeseen circumstances. Such damaged elements require repair to restore their performance characteristics and increase their service life. As a rule, to restore such structures, polymer-based synthetic compounds are used, which are injected into cracks. The injection of calcifying bacteria may also be relevant for the environmentally friendly and autonomous repair of damaged concrete elements. The biological restoration of concrete can be carried out not only by including bacterial spores and a nutrient medium into the concrete mixture in advance, but also by introducing bacterial cells and a nutrient medium directly onto the surface of cracks in concrete elements. This method was used in a study [134], in which the authors used a sprayable bio-based repair compound to repair a damaged car parking ramp. Compared to the control sample, the treatment of the damaged surface led to an increase in the restoration of the load-bearing capacity by 48%.

## 6. Effect of an Autonomous Bacterial-Based Reducing Agent on the Properties of Concrete

Changes in the pore structure significantly impact the diffusion kinetics, which is one of the primary factors affected by the biomineralization process in concrete. The diffusion of moisture and ions, resulting in material corrosion, has beneficial implications for concrete characteristics. Additionally, the process of calcium carbonate formation within intergranular voids facilitates the consolidation of grains, leading to a more densely packed concrete matrix and consequently enhancing concrete strength.

Technologies for precipitation of calcium carbonate using biological agents have made it possible to increase the compressive strength of structural concrete. In the early stages of hardening, thanks to the nutrient medium and the porous structure of the cement mortar, calcifying microbes receive enough nutrients. However, due to the higher pH of the cement slurry, it is possible that bacteria that grow slowly during the initial curing period will adapt to the higher pH level as the concrete cures. During the development of a bacterium, calcium carbonate is deposited on the surface of its cell wall, as well as in the cement mortar matrix, which may be due to the entry of various ions into the environment. This helps to reduce the porosity and permeability of the cement mortar. A significant reduction in the porosity of the matrix leads to blocking access to the concrete matrix of oxygen, moisture, and nutrients. In such a deficiency of the substances necessary for normal life, bacteria either die or go into a spore-like state. This mechanism causes a certain increase in the strength of concrete with the addition of calcifying bacteria [135,136,137,138,139,140,141]. When *Bacillus megaterium* were introduced into a concrete mixture, it was found that the precipitation of calcium carbonate in high-quality concrete was higher than in low-quality concrete. Thus, concrete of a higher strength grade shows a greater increase in strength due to the metabolic activity of bacteria, compared to concrete of a low strength grade. For the highest-grade concrete mixture in the study, 50 MPa, the maximum increase in strength was 24% [142]. By replacing 10% of the cement with fly ash and adding 105 CFU/mL of *Sparcious pasteurii* bacteria, and the subsequent deposition of calcium carbonate on the surface of the bacterial cell wall, a 20% increase in the compressive strength of concrete was recorded [18]. A study [19] showed that the biomineralization of calcium carbonate crystals increased the strength of silicate concrete.

The presence of calcium carbonate in the concrete matrix in this study was determined by X-ray diffraction and electron microscopy methods [143]. The results of the study [144] showed that the compressive strength of concrete with the addition of bacteria *Sparcina pasteurii* and Bacillus subtilis in quantities from 2 to 109 CFU/mL is 20% higher than the compressive strength of concrete without bacteria at the age of 28 days [47]. Based on the results of a study [112], the authors concluded that when replacing cement in a mortar with fly ash with 10, 20 and 40%, bacteria provides an increase in strength by 19, 14 and 10%, respectively, compared to control samples [145]. The authors of [47] added calcifying bacteria to concrete along with the addition of graphite nanoplatelets to ensure a uniform distribution of bacteria in the concrete matrix and, as a result, increase their healing ability. Due to the biomineralization of calcium carbonate by the bacteria Bacillus subtilis in concrete with the addition of graphite nanoplatelets, the compressive strength of concrete increased at all age stages. Many studies [110,146,147] state that the compressive strength of concrete with the addition of a combined additive of bacterial spores increased when the concrete hardened for 28 days. As part of a study [118], alkaliphilic aerobic bacteria were added to the cement mortar along with mixing water in combination with a nutrient medium of urea and calcium chloride. The control composition without the addition of bacteria showed a strength of 55 MPa after 28 days of hardening. The solution with the addition of bacteria showed a compressive strength of about 65 MPa. The authors [54] concluded that the compressive strength of the solution increased by 17% after 7 days of hardening and by 25% after 28 days of hardening, as a result of the biocementation of *Shewanella* species bacteria. The authors of [54] used the bacteria *S. pasteurii* to ensure the biocementation of mortar. It was found that there was no significant difference between the tensile strength of the control sample and the sample containing bacteria, and the readings were 7.78 and 7.45 N/mm^2^, respectively. However, the compressive strength of the sample containing bacteria was higher. When using the bacteria *Bacillus pseudofirmus* and *Bacillus cohnii*, the authors of [109] obtained an increase in the strength of the 28-day solution by 10% compared to the control composition. The authors of [42] studied the compressive strength of the solution using industrial by-products, such as lactose mother liquor and corn extract, as a breeding ground for bacteria. The biocementing bacteria *S. Pasteurii* were used as a biologically active additive. When using lactose mother liquor, the compressive strength increased by 17% after 28 days of curing. The use of corn extract as a breeding ground for bacteria caused an increase in compressive strength by up to 35% [148]. The effect of the biomineralization process of *Arthrobacter crystallopoietes* bacteria on the strength of concrete was also studied by the authors [149], who concluded that this is a promising technology. The authors of the study [150] obtained an increase in strength for concrete containing the bacteria *Bacillus subtilis* by 28% compared to the control composition. According to the authors, the bacteria received nutrition in the cemented material through the absorption of the nutrient medium located in the pores and surrounding space. In this case, during the process of biomineralization, calcium carbonate was deposited both on the surface of the bacterial cell wall and in the cement–mortar matrix. After the pores were clogged with calcium carbonate crystals, the bacteria were deprived of oxygen and either died or were transformed into endospores and acted as microscopic organic fibers [44]. In addition to biomineralization, in general, the increase in the compressive strength of concrete is also associated with the presence of sufficient organic materials in the concrete matrix in the form of microbial biomass, as well as the compaction of voids in cemented materials using biologically derived calcium carbonate. Due to the fact that the origins of the study of the application of the biomineralization process in technical industries lie in the study of its use to improve the physical and mechanical properties of granular soils [151], most of the research on the application of the biomineralization process in concrete is devoted to the study of the mechanical features of the autonomous recovery of concrete at the macro level. A number of studies have shown that when calcite-forming bacteria are not protected, the compressive strength of concrete after the microbial precipitation of calcium carbonate increases by 20–40% compared to reference samples. The authors of [152] found that the use of cementitious materials with a low alkali content to protect the biocementing agent resulted in a strength recovery coefficient approximately 2.3 times higher than in control samples after 28 days of hardening. The authors [123] found that bacteria trapped in lime restored the compressive strength to 50%, which was higher than the control samples, which was about 42%. The authors [25] obtained a recovery of compressive strength of about 60% when using bacteria in polyurethane microcapsules, compared to a recovery of about 5% for bacteria in silica gel microcapsules, which, according to the authors, is due to the higher binding force between polyurethane and the wall of the crack. In [153], the authors obtained an 85% restoration of compressive strength after 28 days of curing using bacteria protected by a coarse aggregate from industrial waste. The authors of [154] also used lightweight coarse aggregate impregnated with 15% calcium lactate and containing bacterial spores to restore a cementitious composite. As a result, the material completely restored its deformability due to bacterial self-healing. The authors of the study [155] obtained a moderate increase in strength by including dead and living bacteria in the mortar without a nutrient medium. The authors suggested that this increase in strength is not due to the process of biomineralization, but rather depends on the stimulation of the deposition of hydration products by bacteria or on the nature of the bacterial cell walls containing some calcium carbonate on the surface.

Given that bacteria and a nutrient medium are introduced into the concrete mix during the mixing stage, it can be inferred that this additive will influence the concrete’s hardening duration. The nutrient medium for calcifying bacteria is usually represented by calcium lactate, calcium nitrate and calcium formate. The introduction of calcium lactate can slow down the curing time of concrete, while calcium nitrate and formate speed it up [44]. The authors of [18] initially studied the influence of “melamine-based microcapsules” on the fresh cement–sand mortar performance. A second peak of hydration in concrete with microcapsules developed more slowly than in the control composition, but, on the other hand, the cumulative heat release after 7 days for both compositions was almost the same. In a study [19], the authors studied the effect of several encapsulating materials on the characteristics of the solution. The inclusion of diatomaceous earth or metakaolin resulted in a decrease in the initial setting time of the solution to 100 min and the final setting time to 250 min. Conversely, the introduction of air-entraining additives into the mixture led to an increase in both the initial and final setting time, from 200 to 240 min and from 300 to 360 min, respectively. The authors of the study [115] used the electrical conductivity method to demonstrate that adding bacteria and growth media directly to concrete slows down the final setting time of cement mortar.

The concrete durability is largely characterized by its resistance to water penetration, as it prevents the penetration of aggressive materials that destroy the concrete. The permeability of binders is usually based on an open-pore system and depends on their tortuosity, cohesion, and specific surface area, as well as the overall porosity of the material and the size of micro-cracks [156]. The biomineralization process has demonstrated the ability to significantly reduce water infiltration into building materials by plugging open pores with calcium carbonate crystals. The authors of the study [41] used a calcium carbonate of microbiological origin, namely “Biocalcin”, during the restoration of a historical building, which caused a fivefold decrease in water absorption in stone elements without changing their aesthetic characteristics and appearance [49]. Similar studies of the effect of the biomineralization of *B. sphaericus* bacteria were carried out in [52] on cement mortar. The authors concluded that the water absorption of the mortar decreased by 60–90% due to the formation of calcium carbonate on the surface of the samples, which prevented the penetration of water into the pores of the material.

The findings of [53] indicate that bioprecipitation of calcium carbonate by *B. sphaericus* caused a noticeable decrease in the water permeability of self-healing bacterial concrete. At the same time, calcium carbonate crystals and bacterial biomass were present both on the surface and inside the porous matrix. The authors of [100] identified threshold-sealing components of coating systems. According to them, the cement surface was sealed by bacteria, which formed a biofilm. Since the bacteria within the biofilm attracted the positive charge of neighboring metal ions, they acted as a center for the formation of the carbonate coating [95]. An increase in the level of urease and carbonic anhydrase enzymes leads to saturation of the liquid phase relative to calcium carbonate, which leads to the deposition of calcium carbonate crystals in the biofilm. The study [19] also confirms a decrease in water permeability due to the formation of a layer of calcium carbonate of biological origin on the surface. The depth of water penetration was greatly reduced in 150 mm cube samples made of bioconcrete with specially bred bacteria of the species *S. pasteurii*. The authors measured the water permeability of the top and side faces as penetration depth. The infiltration at the sides was greater than at the top. The water permeability at the top was 4 times less than in the control samples, and at the side faces—2.5 times. The authors concluded that biocement is designed in such a way that the permeability of concrete is low, since a dense interfacial zone is formed during the biomineralization process. Permeability determines the penetration of aggressive compounds that destroy concrete under the influence of a pressure gradient and is the main parameter that determines durability. The factors determining permeability, in turn, depend on the control of the water–cement ratio, particle size distribution, the age of the set cementitious materials, and the penetration of degrading components. Concrete added with fly ash and *S. pasteurii* bacteria showed a decrease in porosity and permeability, as stated in a study [116]. The water absorption of bioconcrete samples with a bacterial content of 105 CFU/mL decreased by three times [17]. Cube samples prepared with the addition of *Bacillus megaterium* bacteria and a nutrient medium absorbed three times less water than control samples due to biomineralization [157]. The addition of *Bacillus aerius* bacteria reduced water absorption and porosity through biomineralization, which the authors concluded increased the durability of concrete [158]. Bioconcrete samples, due to the clogging of open pores with calcium carbonate crystals of biological origin, showed a significant decrease in water permeability during hardening, while control samples showed a decrease in water permeability during hardening to average values [159]. The precipitation of calcium carbonate by bacteria into the concrete matrix also reduces the water absorption of recycled aggregates, significantly improving their performance.

The most common environmental effect on concrete is the penetration of chloride ions, estimated by charge passing. Typically, it leads to the corrosion of steel reinforcement in concrete, contributing to the weakening of reinforced concrete structures. The resistance of concrete to the penetration of chloride ions can be increased by adding calcium-forming bacteria to the mixture. The average permeability of chloride ions in bioconcrete was 11.7% lower compared to conventional concrete without bacteria. It has also been noted that the addition of certain species of bacteria to concrete, namely Sparcious pasteurii and *Bacillus subtilis*, reduces the volume of concrete susceptible to sulfate formation [115]. The addition of Bacillus aerius bacteria to concrete can reduce the overall charge passing through a control sample of bioconcrete containing rice husks. In a sample of bacterial concrete, charges decreased by 55.8% at the age of 7 days, 49.9% at the age of 28 days, and 48.4% at the age of 56 days [17]. The addition of Sparcious pasteurii bacteria and 10% silica to concrete resulted in the excellent quality resistance of the material to chloride penetration [19]. In a study [95], the greatest mitigation regarding the penetration of chloride ions was observed in the case of concrete samples with the addition of fly ash and bacteria of the genus *Sporoscarcina* in an amount of 105 CFU/mL. However, concrete with 30% fly ash showed very low infiltration. The durability of concrete structures exposed to marine environments or deicing salts is determined effectively by their resistance to chloride ion penetration [160]. The corrosion of reinforcement caused by chlorides is one of the main causes of the destruction of building structures. The high alkaline environment of concrete leads to the passivation of the surface of steel reinforcement, which protects it from corrosion. However, this environment can be neutralized by environmental influences, such as carbonation, causing chlorides to come into contact with the steel reinforcement, causing corrosion. The penetration of chloride ions may be limited by the sealing of pores as a result of biomineralization. However, in the technology for creating self-healing concrete with a bacterial reducing agent, chloride salts are often used. For example, researchers often use calcium chloride as a nutrient medium for bacteria. It is necessary to check the effect of chloride ions present in concrete on steel reinforcement. Researchers often turn to calcium-containing, chlorine-free culture media alternatives. Calcium nitrate can be used for these purposes, as reported in a study [28], where it was productively used as a source of calcium for *S. pasteurii* bacteria. Calcium chloride also causes a massive formation of ammonia, which increases the corrosive effect on steel reinforcement. The use of calcium lactate, as stated in the study [161], when used by bacteria during their metabolic processes, does not lead to the formation of significant amounts of ammonia. In addition to the above, calcium lactate, as stated in the study [28], when used as a nutrient medium for the bacteria *Bacillus cohnii*, led to the formation of large volumes of calcium carbonate crystals ranging in size from 20 to 80 μm on the surface of cracks to heal them. The authors of [162] compared the formation of calcium carbonate by *B. cohnii* bacteria from a nutrient medium of “calcium lactate and calcium glutamate”. A greater thickness of calcium carbonate deposits was observed when calcium glutamate acted as a nutrient medium. Also, the formation of calcium carbonate and urea content for Bacillus species were monitored by the authors of the study [39], using various calcium sources such as calcium chloride, calcium oxide, calcium acetate and calcium nitrate. In this study, the greatest calcium carbonate precipitation was observed when calcium chloride was used as the culture medium. The authors of [50] noted the production of calcium carbonate by bacteria on the surface of mortars reduces capillary water absorption and hydraulic permeability. The existence of bacterial biomass significantly reduces the gas permeability of cement mortar and leads to an increased resistance to carbonization [49]. A carbonate layer thickness of about 30 to 50 µm contributed to increased carbonation resistance. The authors hypothesized that the protective effect of the calcium carbonate coating deposited by bacteria could be further enhanced by external treatment with bacteria and a nutrient medium in the form of calcium sources or increased levels of calcium ions. The authors of [49] measured the resistance of samples treated with bacteria to the penetration of chloride ions using a rapid test. Treatment with biological sediments based on biomineralization led to a decrease in permeability coefficients by chloride ions by 10–40% compared to untreated samples. In addition, the increased resistance to chloride ion penetration in the bacteria-treated solution was similar to the acrylic coating with water-repellent silanes and silicones, and was higher than that of a mixture of silanes and siloxanes. A study [100] evaluated and monitored the effectiveness of biomineralization for chloride ion infiltration in cylindrical cellular bioconcrete samples using a rapid chloride ion permeability test. The bacteria *S. pasteurii* was added to the concrete as a reducing agent. The results were compared with control samples containing no bacteria. The average charge passed through the control samples was 3177 C, while for bioconcrete it was 1019 and 1185 C. Biomineralization reduced the permeability of concrete from moderate to low, according to ASTM C1202-05. The study authors concluded that the use of biomineralization in concrete can be a superior, environmentally friendly and cost-effective sealing technology that can significantly improve the durability of materials.

Calcium carbonate deposits of bacterial origin, according to many researchers, may be an environmentally friendly, innovative way to protect steel reinforcement from its corrosion in concrete. In a study [66], the authors studied the acid resistance of a layer of calcium carbonate of biological origin to sulfuric acid solutions with different pH. The studies were carried out on prototypes in the form of cubes measuring 30 mm. The bacteria of the species *S. pasteurii* were used as calcium-forming bacteria. Since calcium carbonate was deposited onto the surface of the cement mortar by the enzyme urease, produced by bacteria, which further increased the resistance to acid attack, the results showed a remarkable enhancement in the surface’s resistance to the penetration of acid solutions. In their findings, the authors indicated that the layer of calcium carbonate produced by bacteria can withstand acid rain to some extent, which is very useful for protecting steel reinforcement from corrosion and increasing the durability of reinforced concrete structures, especially in coastal areas. The authors of [163] assessed the corrosion resistance of biomineralized reinforced concrete beams. The samples were prepared with the addition of calcifying bacteria in the *Bacillus* genus. Reinforcing bars with a length of 300 mm and a diameter of 25 mm made of steel grade 415 were used as steel reinforcement. In the first two days, the current in the control samples increased from 17.5 to 40 mA, and by the end of the 7 days up to 180 mA was observed, while in the samples from bio-reinforced concrete for the same period, the current was 85 mA. A crack opening width of 0.3 mm is considered the maximum permissible width for reinforced concrete samples. In control samples, such a crack was observed after 36 h, while in bacterial samples, such a crack was observed after 7 days. Taking into account the above results, we can conclude that the biomineralization process can significantly increase the service life of concrete structures. In addition, corrosion current density measurements showed that biomineralization significantly reduced the corrosion current in reinforced concrete samples. Compared to the significantly increased corrosion current in the control samples, which was 60.83 mA/m^2^, in the bioconcrete samples it ranged from 14.78 to 20.03 mA/m^2^. The above-mentioned fourfold drop in corrosion current was due to biomineralization. The authors also concluded that calcium carbonate deposition could promote the formation of a protective passive film and act as a corrosion inhibitor by interfering with corrosion formation in such samples.

Research into the mineralization process suggests that this is a promising and innovative technology that provides long-term storage of atmospheric CO_2_ in the form of carbonates such as calcium carbonate in natural materials, such as magnesite, dolomite, etc. The microorganisms present in such sediments have been studied and characterized [164,165,166]. The processes occurring in them that cause biomineralization were studied in detail [167,168]. The conversion of carbon dioxide to hydrocarbonate requires a carbon dioxide hydration step, which regulates the reaction rate with a forward reaction constant of 6.2 to 10.3 at 25 °C [163]. The “biocatalyst carbonic anhydrase or carbonate dehydratase”, which forms an enzymes group, catalyzes the process of the mutual conversion of carbon dioxide, water, and unbound carbonic acid ions, that is, HCO_3_^−^ and hydrogen. These substances attract the attention of researchers because they play an important role in the calcification of marine invertebrates such as mollusks and corals, as well as in the hard tissues of vertebrates and the otoliths of fish [57]. Initial studies of the possibility of carbon dioxide sequestration using carbonic anhydrases used bovine carbonic anhydrases, which contributed to the study of bacterial sources [169,170,171,172,173,174]. Carbonic anhydrase biomineralization provides the ability “to capture carbon dioxide into a stable, safe, and long-lasting CO_3_^2−^ deposition environment”, thereby becoming a perpetual method for removing CO_2_ from the atmosphere [175,176]. In recent years, it was discovered that bacterial carbonic anhydrase, obtained from large rod-shaped bacteria of the species *Bacillus megaterium*, acts synergistically with urease during the deposition of calcium carbonate [177]. The ability of this enzyme to produce a precipitate of calcium carbonate crystals from a calcium-containing nutrient medium is realized with the help of chemical buffers and pure CO_2_ gas [149]. Following the formation of biominerals through the metabolic processes of bacteria, a number of researchers have identified species such as Cyanobacteria or Cyanophyta, which could be used as effective mechanisms for capturing and storing carbon using solar energy for photosynthesis, resulting in CO_2_ being converted into a stable mineral form—calcium carbonate [178]. These particular bacteria are classified as photosynthetic prokaryotes, deriving energy through the process of photosynthesis and having the ability to produce oxygen. Biomineralization has the ability to accelerate the release of dissolved inorganic carbon into the environment, thus enabling the retention of carbon. This procedure accomplishes this by ensuring the availability of cations in a solution while simultaneously preserving pH [179]. In the natural environment, these cations can be easily obtained through the evaporation of sediments, wastewater, saline aquifers, oil waste, and abundant seawater reserves.

## 7. The Influence of Additives in the Form of a Nutrient Medium for Bacteria and Microcapsules on the Concrete Performance

Adding a nutrient medium for bacteria to a concrete mixture can affect the performance of the final material by changing its microstructure [118,120,180]. It is also impossible not to note the fact that when adding a bacterial self-healing agent protected by microcapsules, it is inevitable to encounter such factors as the low adhesion of the concrete matrix to the microcapsule material, as well as the formation of voids in the concrete when the microcapsules release their contents. The formation of such voids can significantly affect the strength of concrete and its other mechanical and deformational properties. The introduction of bacterial spores directly into concrete is associated with losses of about 8–10% of compressive and flexural strength [40]. The authors of the study [28] directly included spores of the bacteria *Bacillus cohnii* and *Bacillus pseudofirmus* into the concrete mixture, which led to a decrease in strength by approximately 10% when the concrete was 28 days old. The authors of [181] prepared a cyclically enriched ureolytic powder for mortar, which was added to the mixture in amounts of 0.5% and 1% by weight of cement. When introducing this additive, no negative effect on the compressive strength of the solution was noted. However, a higher dosage of this additive, 3% and 5% by weight of cement, led to a significant decrease in strength. The study [24] reports experimental results showing a significant effect of nutrient additives and microcapsules on hydration, as well as on compressive and tensile strength. When adding 5% microcapsules by weight of cement, the compressive strength was reduced by 34%. The addition of more than 3% microcapsules by weight of cement also affected the tensile strength. The addition of a nutrient medium for bacteria had less effect on the physical and mechanical characteristics of concrete than the addition of microcapsules. The degree and rate of hydration, as a rule, improved, but in the case when calcium nitrate was used as a nutrient medium for bacteria, microcapsules and yeast extract, on the contrary, hindered the hydration process. In this case, calcium nitrate can be considered as a process-accelerating additive, so its dosage should be carefully calculated. The yeast extract in this case slowed down the hydration reaction by preventing the cement particles from reacting with the water. The authors of [23] confirmed a significant decrease in strength when replacing granite crushed stone in concrete with a lightweight aggregate containing spores of calcifying bacteria and a nutrient medium. Lightweight aggregates are more easily destroyed under load, thereby quickly starting the process of autonomous recovery, releasing bacteria and a nutrient medium. However, this type of concrete has lower strength and cannot be used as a material for load-bearing structures. It is also known that in cases where only calcium lactate was used as a mineral nutrient medium, instead of a decrease in strength, a slight increase in strength was observed [23,28,40]. The addition of yeast extract and peptone reduces the compressive strength of concrete, especially in the late stage of hardening [28]. Microcapsules, when added to a concrete mixture, caused a decrease in the water absorption of concrete [24]. The decrease in strength was mainly due to changes in the microstructure of the material due to a decrease in the degree of hydration and poor distribution of hydration products, which in turn was due to the combined effect of nutrients and microcapsules.

Analyzing the conclusions drawn from the above studies, it can be noted that water absorption depends on the degree of hydration and the number of open pores in the concrete matrix. If the nutrient medium for bacteria has a waterproofing effect, microcapsules can clog some of the open pores, because of which, despite a general decrease in strength, an increase in the durability of concrete will be observed due to a decrease in water absorption and other characteristics dependent on the porosity of the material. The addition of microcapsules can also affect the rheological properties of concrete, which must be taken into account when creating self-healing bioconcrete with a biological reducing agent protected by microcapsules. Rheological characteristics may depend on the capsule material, shape, and size. Spherical microcapsules can have a lubricating effect by reducing the adhesion of cement gel to aggregates [182]. A decrease in viscosity and a decrease in yield stress were also noted. However, the improvement of rheological properties will depend on the microcapsule material, which should not be too absorbent, so as not to absorb part of the water in the mixture, which should be involved in hydration reactions, and also affects the shrinkage and flow of the fresh mixture [183].

## 8. The Physical and Mechanical Properties of Self-Healing Concrete Restored Using a Bacterial Reducing Factor

Restoring the original properties of concrete will depend on the degree of its damage, namely on the width of the opening and the depth of penetration of healed cracks. Also important are factors such as the strength of the material used to fill the cracks and its adhesion to the material matrix and its structure. An effective autonomous recovery process requires a high rate of restoration of the original properties of the material. The autonomous recovery of self-healing bioconcrete is determined by factors such as the curing conditions, the local concentrations of viable spores and nutrients, the age of the concrete, and the healing time. Bacterial self-healing concrete is capable of processing a wide range of cracks, but the effectiveness of self-healing concrete is determined by the degree to which its mechanical properties and durability are restored.

The authors of [25] encapsulated bacterial cells in laboratory conditions using polyurethane and silica gel, where the width of the healed crack was 0.35 mm. When the microcapsule material was silica gel, an increased amount of calcium carbonate crystal precipitation was found, but the strength recovery was about 5%. In the case when polyurethane acted as the microcapsule material, a recovery of strength from 50% to 80% was observed. When using silica gel microcapsules, the amount of calcium carbonate crystal formation was greater than in samples with polyurethane microcapsules, while strength recovery was higher in the latter samples. In [26], the authors found that the inclusion of calcifying bacteria in concrete increases its strength through three possible mechanisms. Carbon dioxide, which is produced by the metabolic processes of bacteria, reacts with the portlandite on the surface of the crack, turning into a stable and insoluble form of calcium carbonate. This calcium carbonate is responsible for reducing porosity and increasing particle packing capacity, which leads to increased strength [184]. One way or another, negatively charged bacteria can become centers of cement hydration. Despite the strength of calcium carbonate, it should be noted that the strength recovery resulting from its binding to the concrete matrix may be limited.

The effective self-healing of concrete should, ideally, completely or almost completely restore the original durability and strength of the material. Durability is often assessed by properties such as water permeability and water absorption. Healing cracks involve blocking the pores with certain chemicals, preventing the penetration of air or water. The decrease in water permeability is entirely due to the blockage of pores by water-insoluble calcium carbonate produced by bacteria [184]. The authors of the study [185] concluded that the healing coefficient decreases, from 83% for a crack with an opening width of 0.1–0.3 mm, to 30% for a crack opening width of 0.8 mm. The authors attribute this to the fact that at large crack opening widths, a sufficiently large amount of healing material is washed off from the concrete surface due to the entry of moist air and water into the crack. Also, a decrease in the healing coefficient may be due to the lack of the required amount of nutrient medium at the site of damage or to the loss of sealing material for other reasons [40]. The process of autonomous bacterial repair is directly related to the metabolism of bacteria and the formation of calcium carbonate crystals can be limited by their concentration, as well as the type of starting material and nutrients at the site of formation of the healed crack. When the rate of formation of calcium carbonate crystals is low, there is an increased risk that the deposited sealing material may be washed out of the crack before it is completely healed. A number of researchers confirm the fact that, in experiments, samples immersed in water during autonomous recovery have a higher healing coefficient than when samples harden in wet conditions [15]. This phenomenon may be due to a better transport process of the healing agents due to the existing concentration difference in the material matrix and on the submerged surface. The study authors also noted that the best autonomic recovery rate during wet curing was achieved at an early age of the material due to the fact that at this stage a sufficiently large amount of water is available for bacterial activity. The rate of healing during wet curing was slow and was approximately equal to that of a late-stage immersion cure. The authors of [24] obtained a similar result when using *Bacillus sphaericus* bacterial spores protected by melamine microcapsules as a healing agent. A crack with a length of 850 to 970 mm was healed by immersing the prototype in water. However, the healing capacity was higher in the case of alternating wet and dry cycles. The authors attribute this to the fact that in the wet cycle, moisture enters the crack, and in the dry cycle, the bacteria received a large amount of oxygen, which is also necessary for active metabolism. If the samples were completely immersed in water, the bacteria did not receive enough oxygen. It is also important to understand that in the real operating conditions of building structures, complete immersion in water is a rather rare case, and most often, the creation of such conditions is impossible.

The area of the healed crack is a more objective indicator of autonomous recovery compared to the opening width of the healed crack, since the latter indicator is characterized by only one direction, while different lengths of the healed crack may imply completely different amounts of healing. Studies on water permeability have shown that concrete samples with the addition of microcapsules with bacteria in an amount of 5% by weight have a minimum limiting coefficient of water permeability when alternating dry and wet cycles. Moreover, despite the fact that this value was close for samples with a microcapsule content of 3%, much smaller differences in water permeability values were found in samples with 5% microcapsules. The study authors note that concrete with 5% microcapsule content has a lower strength. Thus, in this case, a low permeability value can be interpreted as a decrease in porosity with an increase in the number of microcapsules, but only if the capsules remain intact. After microcapsule rupture, the lower permeability for samples with 5% microcapsule content is likely due to the waterproofing effect caused by the healing material, which seals cracks and micropores. However, the optimal amount of microcapsules, from the point of view of the effect on strength and water permeability, is 3%. The material of the capsules and the type of nutrient medium can significantly affect the drop in water permeability. The authors of the study [25] obtained a twofold reduction in water permeability when using silica gel microcapsules and a more pronounced bacterial effect on clogging pores with calcium carbonate crystals. However, when using microcapsules made of polyurethane, the reducing activity of bacteria was significantly lower, and the authors explained that the improved reduction in water permeability, compared with microcapsules made of silica gel, was a result of the more pronounced waterproofing properties of polyurethane [25]. This waterproofing mechanism is often used when using polyurethane foam and calcium-forming bacterial spores in glass capsules. Calcium carbonate, produced by bacterial metabolism, could reduce the porosity of polyurethane foam and have the least effect on directly sealing concrete pores. A significant reduction in water permeability was obtained by the authors of [30] using hydrogel microcapsules that protect bacterial spores. The largest opening width of a healed crack was 0.5 mm; however, even for cracks with a smaller opening width, such as 0.3–0.4 mm, there was a strong discrepancy in the healing coefficient, which amounted to 40–90%. The proportional distribution of bacterial spores and culture medium when encapsulated in a hydrogel may result in improved healing. Also, the ability of the hydrogel to absorb and retain moisture will play a positive role in this case, which can increase the activity of bacteria. In addition to the formation of calcium carbonate crystals, a decrease in water permeability may also be caused by the internal curing system of the hydrogel. The authors of [29] noted a noticeable improvement in water absorption with the addition of bacteria encapsulated with diatomaceous earth. Water absorption decreased by 1/3 and 50%, and damaged samples were kept at high temperatures in calcium nitrate, yeast, urease and water. Calcium ions and urea from the environment penetrated into the crack and increased the formation of calcium carbonate during self-healing. The cracking of the prototypes, however, was carried out in samples aged 14 days, which allowed nutrients and calcium to be easily transferred from the environment to the concrete matrix. The study’s authors [29] also observed that an increased calcium ion content results in a reduction of “hydroxyl ions from calcium hydroxide dissolution”, ultimately causing a pH decline. When late-stage concrete, which possesses a dense microstructure resulting from the hydration process, is submerged in a nutrient medium, the penetration of calcium ions into the concrete matrix becomes hindered. Therefore, the concentration of calcium ions will only be increased on the surface that hinders the exposure of bacterial spores to an external reservoir of calcium and nutrients. This review could be applicable in evaluating the dependability of self-healing cementitious composites through the implementation of the reducing agent encapsulation technique. It encompasses various aspects such as the strength, reliability, availability, durability, quality, and versatility of the construction material, in accordance with the provisions outlined by the authors [186].

## 9. The Features of the Macro-, Micro- and Nanostructure of Self-Healing Bioconcrete

The results of studying concrete and mortar using electron microscopy methods showed the formation of calcium carbonate crystals in the material matrix during the metabolism of rod-shaped bacteria. The growth of calcium carbonate crystals in concrete increased its impermeability as they plugged pores and micro-cracks, thereby preventing harmful materials from penetrating into the concrete matrix [116]. The introduction of calcium-forming bacteria into a concrete mixture improves its microstructure due to the compaction of calcium carbonate crystals formed during biomineralization, which was confirmed by the results of studies using electron microscopy, X-ray diffraction and electrodispersive spectroscopy. According to a study [18], the addition of *Bacillus megaterium* bacteria at a concentration of 30 to 105 CFU/mL produced the highest mass of calcium carbonate, about 38.76%, compared to other proportions of the mixture with and without bacteria [17]. Electron microscopy analysis revealed heterogeneous calcium carbonate crystals on the bacterial cell walls. It was noted that calcium carbonate crystals contained large amounts of calcium in the sample, which was confirmed by X-ray diffraction and electrodispersive spectroscopy. This structure can improve the durability of concrete [19]. Micrographs of bacteria-free reference samples as well as bacterial concrete samples were analyzed. The analysis confirmed the formation of calcium carbonate crystals in bacterial concrete [185,187]. The addition of bacterial amalgamation led to an enhancement in the compressive strength of rice-husk-ash-infused concrete, attributable to the formation of calcium carbonate crystals within the pores. This was verified through micrographs of the specimens. Analyzing the above, we can conclude that calcium carbonate crystals clog the pores and voids in bacterial concrete, completely filling them. The deposition of calcium carbonate crystals inside cracks when testing samples was confirmed by the results of a microstructure study. Consequently, water absorption, permeability to chloride ions, and acid infiltration were reduced due to compaction of the microstructure, which was shown to increase the signal transmission rate when examining concrete using the ultrasonic method [19]. Typically, studies of this nature are conducted on a small scale in order to detect deposits within cracks in concrete samples after their healing, thereby enhancing the reliability of the obtained outcomes. Due to this rationale, the majority of researchers have utilized electron microscopy, field emission scanning microscopy, and X-ray diffraction. Electron microscopy allows one to recognize the morphology of materials inside cracks in concrete [17]. Precipitated biomineralization products are calcium carbonate crystals formed by various types of bacteria mixed with hydration products. Relatively recently, the process of autonomous bacterial recovery was studied using Raman spectroscopy [188]. Research has shown that the use of calcium-forming bacteria as a reducing agent in concrete is an extremely reliable technology [189]. Researchers have conducted some macro-level studies in terms of autonomic recovery processes [189]. These include impact strength testing and axial tensile testing [54,190]. Studies were also carried out using the ultrasonic method and water permeability studies [191].

Several researchers have conducted additional tests on sorption capacity and gas permeability to test the effectiveness of the autonomous reduction of sodium silicate, colloidal silica, and tetraethyl orthosilicate [192,193]. The study revealed a reduction of 18% in gas permeability and 69% in sorption capacity. The authors [165,169,170,171,172,173] conducted a hardness study to test and evaluate the characteristics of the material after bacterial self-healing. A study [74] shows that the flexural strength of concrete after healing was 50% greater than that of the control sample. The authors of [194] performed a thermogravimetric analysis to identify white deposits inside the crack. A number of researchers have devoted their work to chloride ion penetration, oxygen profile, porosity, and pore size distribution. Regarding compressive strength, the study authors emphasize that it can be restored up to 60% through an autonomous recovery process. An acceleration of the signal during ultrasound examination after self-healing was also noted, which indicates the compaction of the material. Taking into account the research results, it can be said that durability problems with more important impacts over a long period can be solved using a biological approach to self-healing based on microbial activity. This technology can extend the service life of concrete and reinforced concrete elements. However, studies of the adhesion force between materials deposited inside cracks in concrete, as well as studies of gas permeability and deformability, are very few and practically non-existent. Therefore, it is necessary to evaluate the improvement of self-healing bioconcrete using different approaches. X-ray diffraction studies revealed a combination of biomineralized sediments including calcite, aragonite and vaterite. X-ray diffraction results were compared for samples cured under wet–dry and completely wet conditions. The X-ray diffraction spectra of bacterial concrete revealed a higher amplitude of peaks during the wet–dry curing phase as opposed to the completely wet curing phase. “Aragonite had distinct peaks and maximum peaks for the wet-dry-cured and all-wet-cured samples at 28.511 and 38.447, respectively, while calcite showed peaks at 20.298, 25.163, 26.208, 27.358, 47.612, 49.256, 55.605, 56.481, etc. d”. It is important to note that a large amount of calcite was found in samples of bacterial concrete, which is responsible for the decrease in water absorption of the concrete mixture [195].

To increase the reliability of tests, a number of authors have conducted research on the nanoscale. The authors of [39] assessed the self-healing ability using nanostructure studies. The average nanomechanical values of the transition zone are 20% higher than those of the previous precipitates, which served as a strong bond between the concrete and the precipitated layer of calcium carbonate. It is advisable to conduct bond strength tests at macro- and nanoscale levels to check the interface between the deposit layer of the repair material and the cement gel inside cracks in concrete [196]. The authors of [197] introduced a new autonomous recovery scheme based on micro- and nanoparticles. The microparticles were silica microcapsules containing epoxy sealing compounds. Silica and functionalized amine acted as nanoparticles.

## 10. Impact on the Environment

One of the most important issues in the development of any technology is its safety. This is especially important when working with bacteria. This characteristic is certainly considered by the developers. All the bacterial species mentioned in this review belong to the most pathogenically safe «group 1» according to the classification adopted, for example, in the EC [198]. However, when developing commercial biopreparation (supplement) for concrete and putting them on the market, it will be necessary to undergo safety confirmation procedures established by the laws of various countries.

The biomineralization process on which the technology for creating self-healing bioconcrete is based is generally considered to be very sustainable due to its economic and energy efficiency, as well as its recyclability. Despite this, the application of this process in the field of mass production of individual building materials is still difficult and far from practical implementation. However, the use of biocement produced through the biomineralization process as an additive in the repair and restoration of building materials has shown promising results in terms of sustainable construction. Research into the application of the biomineralization process in building materials shows that it can significantly improve the properties of popular and widely used building materials, such as sand, limestone, concrete and mortar. The ability of the mineralization process to increase the strength and durability of concrete, while reducing the permeability, is the most promising factor in terms of sustainability. By reducing the need for the amount of material due to an increase in its strength and durability, and, consequently, service life, the stability of such a material also increases [199]. The life-cycle assessment methodology simplifies the process of assessing environmental impact and is one of the key points to confirm sustainability aspects. A number of studies have identified the impact of technological advances in recent decades on cement, concrete and the environment [200,201,202,203,204]. One study demonstrated the conceptual importance of the life-cycle assessment steps of defining goals and scope, the life-cycle inventory, and the life-cycle impact assessment. New trends in the use of the life-cycle assessment methodology involve accepting that for cement strength, life-cycle assessment results are influenced by more than 10%. The importance of determining strength properties and durability associated with functional units in determining the life cycle of concrete has also been noted [153]. The life-cycle assessment method can help to study the environmental impact of the biomineralization process.

Currently, despite the promise of this approach, the number of such studies is limited by problems such as the lack of a precise definition of system boundaries, the inadequacy of data, providing a limited understanding of life-cycle indicators, as well as service life. Further research and analysis, even of the effects of urea and calcium chloride use, is also required [205]. These research gaps will be filled in the coming years by researchers in the fields of biomineralization process and life-cycle assessment. However, some preliminary conclusions can already be drawn regarding the life-cycle assessment for the biomineralization process. The availability of reproducible and reliable data for the life-cycle assessment may be limited in the near future due to the lack of commercial applications. Consequently, life-cycle studies will probably start with local and limited available data. Functional life-cycle assessment units are typically used for presentation purposes such as strength and durability. Units such as mass, volume, etc., should not be used. A reliable assessment of the durability and service life, as well as performance characteristics of concrete, will greatly contribute to the emergence of reliable life-cycle assessment studies. The use of industrial by-products as a source of calcium, urea, and nutrients for bacterial growth can significantly reduce the environmental impact of the biomineralization process, as well as increase its economic efficiency, which must be taken into account in life-cycle studies.

## 11. Advantages and Prospects for Further Research on Self-Healing Bioconcrete

The main advantages of using the biomineralization process in cement composites lie in the factors of the compaction of their microstructure due to the clogging of pores and micro-cracks with calcium carbonate crystals, and the collective binding of grains of the loose structure of such materials into a single whole. These factors have been identified and described by researchers, but they are too influenced by the hardening conditions and environmental features. This suggests that self-healing bioconcrete technology has not yet been optimized for use in different parts of the world, and requires scaling up for commercial scale applications. Also important is the issue of developing a regulatory framework for the use of such materials, describing their nomenclature, and creating standard protocols for their testing and control. It is also imperative to solve the issues of reducing energy consumption and sequestering carbon dioxide from the atmosphere, as well as recyclability. However, the above goals require a large amount of research data, which to date still needs to be gathered. Research on the life cycle of biomineralized structures and the materials based on them is also necessary. However, the data obtained to date can already be used to roughly assess the prospects of self-healing bioconcrete. Since the technology for creating bioconcrete is being developed at the intersection of the construction and microbiological industries, certain concerns arise about the possibility of using bacteria in mass construction, where they have not traditionally been used before. First of all, the issue of health safety and economic efficiency is important. Numerous studies on the harm of calcium-forming bacteria to workers have dispelled these concerns. The biomineralization process also expands the possibilities for the recycling and beneficial disposal of industrial by-products. However, the use of by-products requires adaptation to local conditions, in accordance with the location of the source of the waste being disposed of. Also, in addition to the issues mentioned, the biomineralization process has great potential for use in various areas of the construction and infrastructure industry due to a significant reduction in the water and chloride permeability of materials, the possibility of autonomous healing of cracks even with an opening width of up to 0.97 mm and a depth of 32 mm, as well as due to imparting sealing properties to concrete, which makes the use of the biomineralization process in concrete an economically beneficial and environmentally friendly way to increase its durability.

**Figure 6:**
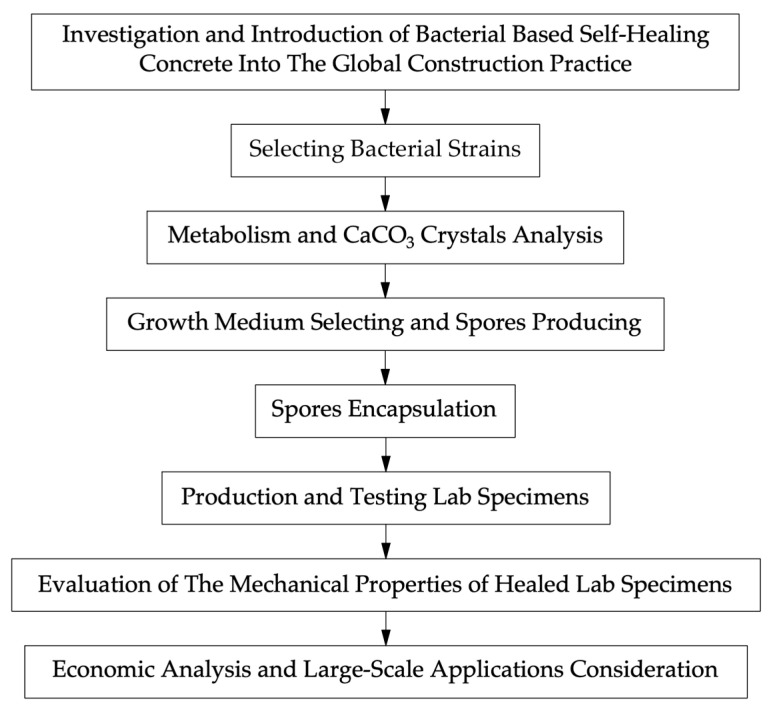
The process of introducing self-healing bioconcrete into construction practice.

However, before commencing the commercial application of self-healing bioconcrete technology, it requires large-scale and comprehensive research, with a continuous filling of the existing and emerging gaps in the process. The problem of nutrient optimization also needs to be addressed. It is necessary to study concrete properties such as corrosion, shrinkage, and carbonation carefully in relation to bioconcrete. The above studies can help identify and characterize the life cycle of such materials and their real-time behavior. So far, the issues discussed above have been studied primarily in laboratory conditions, which makes it necessary to study the potential of autonomous concrete recovery technology based on the biomineralization process under real conditions of the intended use of such a material. Namely, these factors include wide temperature ranges, higher salt concentrations, and the later service life of concrete structures. A detailed study of the process of autonomous bacterial reduction and various methods of its application is the main task for promoting this technology in mass construction. Figure 6 shows the process of developing and introducing self-healing concrete into construction practice.

**Figure 7:**
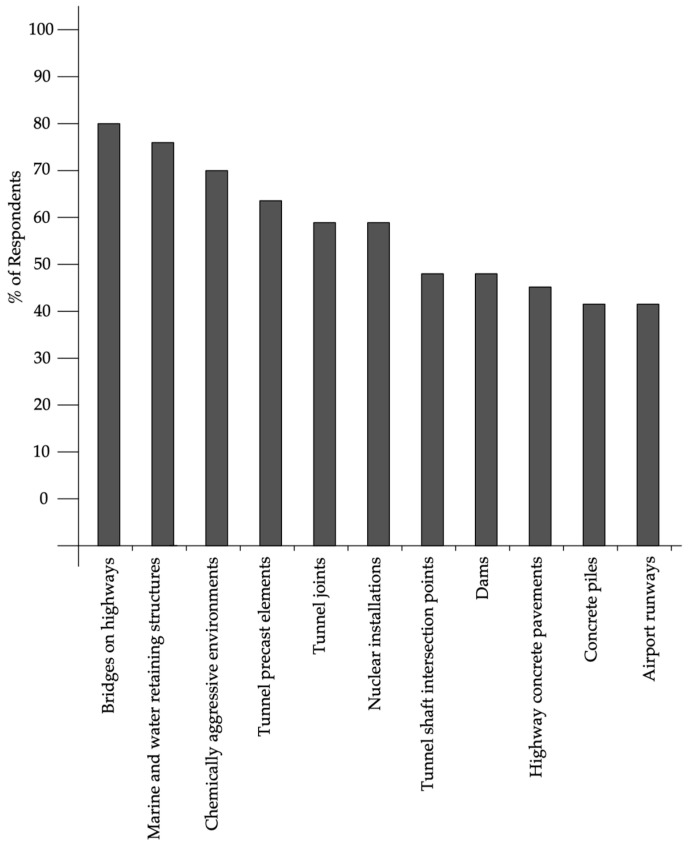
Potential applications of self-healing bioconcrete based on respondents’ opinions.

The full impact of biomineralization technology on the construction industry, despite its significant potential to improve the quality of building materials, can only be assessed at the scale of commercial mass application. The main problem in the development of self-healing bioconcrete technology, from scientific research to mass use in global construction, is the interdisciplinarity of this topic and its lack of information coverage. As a rule, construction industry specialists are far from the field of microbiology, and perceive any bacteria as a potential threat to the health and life of workers. That is, the roots of this problem lie in the informational and psychological factors. Basic knowledge of the microbiology and pathogenicity of bacteria used in self-healing technology would greatly benefit the construction community and the construction industry, as well as the building materials industry. The survival of certain types of calcium-forming bacteria in the highly alkaline environment of cement mortar has been well studied and described in many studies. A number of bacterial species resistant to high pH environments have been isolated. However, the use of technology to protect bacteria using microcapsules and the use of calcium chloride as a nutrient medium for bacteria raises certain concerns among specialists in the construction industry. A number of calcium-containing nutrient medium substitutes without the use of chlorine have been investigated and isolated; however, they are more expensive than calcium chloride. Thus, economic aspects are also important for the popularization of self-healing bioconcrete technology. A good contribution to the cost-effectiveness of these materials can be the use of recycled materials, which can provide benefits both by reducing material costs and by improving the environment. The authors of [109] determined that recycling efficiency depends on industrial by-products and potential contaminants, such as lactose mother liquor and corn extract, which can be used as a breeding ground for bacteria. At the same time, the costs of creating self-healing concrete fell by 70%. Likewise, other components of the biomineralization technology can be replaced with suitable natural or processed substitutes. Figure 7 presents the results of a survey of respondents regarding the potential applications of self-healing bioconcrete.

A total of 700 respondents were interviewed. The survey was conducted in higher education institutions (teachers, students), at enterprises producing fresh and hardened concrete, at construction sites, and on the street with random respondents. The survey formats were face-to-face, via a phone call, and via messengers or social networks. Each respondent provided several options for the potential areas of application of self-healing concrete.

## 12. Discussion and Conclusions

The long service life of concrete leads to the fact that, along with the constantly growing volume of production of new concrete, and reinforced concrete structures and elements, structures built many decades ago also continue to be used. Such structures gradually wear out and, throughout their service life, require a large amount of work to repair and restore them. As a rule, the cost of these works is very high as a result. The author of the study [206], in his work, describes this aspect using the example of the life-cycle cost model he developed. According to his conclusions, in some cases, renovation costs may even exceed the initial construction costs. Autonomous bacterial concrete reduction technology can significantly reduce these costs. The autonomous restoration of bacterial concrete is much more effective when bacteria are encapsulated using microcapsules. There is also very little evidence of the autonomous recovery of fatigue deformations. The recovery of such deformations will largely depend on the behavior of the microcapsules [207]. The controlled, “smart” release of bacteria from microcapsules can lead to the healing of deformations that have occurred over several load cycles. The authors of [208] worked to develop a technology for the controlled release of microcapsule contents to treat deformations in concrete, especially the corrosion of steel reinforcement caused by a decrease in the alkalinity of concrete. However, such research on the implementation of a “smart”, controlled release of the bacterial contents of microcapsules in relation to self-healing bioconcrete has not yet been fully implemented to date. Also promising at the moment is the issue of creating nanocapsules to reduce the size of voids formed due to the inclusion of microcapsules in the concrete matrix. The research using the method of “sonification” of urinary-formaldehyde capsules in self-healing bioconcrete may also be interesting, which have a diameter of 220 nm and a thickness of 77 nm, with a more uniform shell, which has so far been used only for the encapsulation of chemical reducing agents. However, when using such technologies, the issue of the agglomeration of waste nanoparticles in the concrete matrix is important, since they can cause the development of cracks in the material. To date, research on self-healing bioconcrete suggests that the average time required for the healing of cracks in bacterial concrete, subject to the necessary curing conditions, is quite long and is at least 2–3 weeks.

It is presumably possible to speed up the healing process and increase the lifespan of bacteria in self-healing bioconcrete by obtaining new, modified species of bacteria obtained as a result of selection and genetic modification, but this requires serious interdisciplinary research. This introduces the prospect of creating self-healing bioconcrete in the future, capable of healing cracks in a short time, even with a large opening width. Also, a promising and important related direction is the control of the crack opening width [209,210,211]. An example of such a technology is the technology of fiber-reinforced concrete, in which dispersedly distributed reinforcing fibers significantly limit the development of cracks, which in the future can significantly accelerate the process of autonomous healing [212,213,214,215,216,217,218]. Also, many studies consider the process of bacterial healing of concrete under ideal conditions, while it is necessary to conduct research into the process of autonomous bacterial healing under real operating conditions of building structures. The main focuses of such research should be increasing service life, reducing costs, and the social and environmental benefits. A promising aspect is the study of the properties of autonomously recovering bioconcrete from the point of view of adaptation to changing climatic conditions [173]. The life-cycle studies of self-healing materials with bacterial reducing factor may also be relevant for their improvement [219,220]. From this point of view, the most promising technologies are the creation of reducing bacterial agents that sequester carbon, which can make such building materials sustainable and “green” throughout their entire service life [74,221,222,223,224,225,226,227,228,229,230,231,232,233,234,235,236,237,238,239,240,241,242,243,244,245,246,247,248,249,250,251,252,253,254]. It should be noted that the issue of self-healing concrete is very relevant, but it depends on the time factor. Research into the effectiveness of various methods for increasing the durability of self-healing concrete requires long-term verification over several decades. Due to the fact that the topic of self-healing concrete is quite new in the chronology of construction science, at the moment we can only be guided by operational data in the last decade. However, modern methods for predicting the properties of concrete, as well as the quality of the resulting structure and the proven effectiveness of the technologies used, from the point of view of building materials and biological processes, already have a fairly deep, well-developed scientific and technical basis, and it can be assumed that the use of such concrete will lead to an increase in the quality, safety and durability of building structures.

Despite the large amount of research into self-healing bioconcrete, regulations have not yet been developed that would set out specifications, standards and test methods for assessing the autonomous recovery of such materials in building structures. Ultimately, further research should lead to the creation of such a regulatory framework. Analyzing studies of self-healing bioconcrete conducted over the past decade, we can assume the main areas of their application in the foreseeable future. However, this should be preceded by research and work devoted to the optimization and improvement of self-healing bioconcrete, which would make it suitable for mass use and would contribute to the popularization and widespread use of this material.

Despite the large number of studies available in the scientific literature on self-healing concrete, no such large-scale review and analysis has been conducted. Therefore, we have systematized the existing knowledge on this topic.

The present article is devoted to the technology of creating self-healing concrete based on a bacterial reducing agent. It examines in detail both the process of autonomous restoration of such a material and the effectiveness of crack healing as a result of the metabolic processes of calcifying bacteria in restoring the physical, mechanical and deformable properties of concrete. As a result of the analytical work carried out, the following conclusions can be drawn:

1. The formation of calcium carbonate crystals in concrete during biomineralization improves its compressive strength.

2. The high compressive strength of concrete has a positive effect on bacterial activity in self-healing bioconcrete.

3. The results of numerous studies of self-healing bioconcrete using electron microscopy, X-ray diffraction and energy dispersive spectroscopy show that the biomineralization process promotes the compaction of the concrete microstructure due to the growth of calcium carbonate crystals in the material matrix, reducing its permeability to water and chloride ions and, as a result, improving its durability.

4. The addition of calcium lactate media slows down the curing time of concrete, while calcium formate and calcium nitrate can increase it.

5. The most effective bacterial reducing agents for creating self-healing bioconcrete are spores of the bacteria *Bacillus pasteurii* and *Bacillus subtilis*.

6. The bacteria of the species *Sporosarcina pasteurii* and Bacillus subtilis, when used as a reducing agent in self-healing bioconcrete, additionally reduce the penetration of chloride ions into concrete, which increases the sulfate resistance of the material.

7. The use of bacteria of the species *Bacillus cohnii* and *Bacillus pseudofirmus* simultaneously in self-healing bioconcrete as a reducing factor leads to a decrease in strength.

8. Coating the concrete surface with a layer of calcium carbonate, formed during the life of calcifying bacteria, to some extent allows such an element to withstand acid rain, and also effectively protects steel reinforcement from corrosion, especially in coastal areas, thereby significantly increasing the durability of such structures.

## Figures and Tables

**Table 1:** Studies of self-healing lightweight bioconcrete with various types of coarse lightweight aggregate.

Reference	Type of Bacteria Used in the Study	Type of Lightweight Porous Aggregate
[83]	*Bacillus pseudofirmus*	Expanded clay
[84]	*Paenibacillus mucilaginosus*	Expanded vermiculite
[34]	*Bacillus sphaericus*	Diatomaceous earth, expanded clay, granular activated carbon, metakaolin, zeolite, and air entrainment
[85]	*Sporosarcina Halophila*	Expanded perlite aggregates
[86]	*Sporosarcina pasteurii*	Porous and superlight expanded glass
[87,88,89]	Alkaliphilic bacteria of the genus *Bacillus*	Expanded clay granules
[90]	*Sporosarcina pasteurii*	Expanded shale aggregate
[91]	*Bacillus psuedofirmus*	Expanded perlite
[91]	*Lysinibacillus boronitolerans*	Expanded clay
[92]	*Sporosarcina Pasteurii, Bacillus Megateterium, Sporosarcina Ureae* and *Bacillus Licheniformis*	Leca coarse LWA and Leca fine LWA
[93]	*Bacillus mucilaginous*	Expanded perlite
[94]	*Bacillus alkalinitrilicus*	Expanded clay particles
[76]	*Bacillus subtilis*	Diatomite pellet
[95]	*Bacillus subtilis*	Pumice
[96]	*Sporosarcina pasteurii*	Ceramsite particles
[97]	*Bacillus alcalophilus*	Modified ceramsite particles
[98]	*Bacillus mucilaginous*	Ceramsite

**Table 2:** Healing capacity of some types of bacteria in self-healing concrete.

Ref.	Type of Bacteria Used in the Study	Method for Adding Bacterial Spores to a Mixture	Type of Nutrient Medium	Opening Width of Healed Crack, mm	Restoring Strength	Improved Durability
[119]	*B. subtilis*	Diatomite lam dong	Urea, CaCl_2_ H_2_O	1–1.8	−	+
[120]	*B. pseudomycoides*	Directly with 100 mL cell	Ureolytic activity	0.15–0.3	+	+
[121]	*B. subtilis*	Directly with 2.2 × 10^6^ cells/mL	Urea—2CaCl_2_ curing	0.2	−	+
[29]	*S. pasteurii*	Directly with 10^7^ cells/cm^3^	Urea—CaCl_2_ curing	0.28–0.34	+	+
[122]	*B. sphaericus*	Diatomaceous earth with 10^9^ cell/mL	Urea, yeast extract, Ca(NO_3_)_2_, 4H_2_O	0.15–0.17	-	+
[30]	*B. megaterium*	Directly with 2.2 × 10^6^ cells/mL	Urea yeast extract, beef extract	0.3	+	+
[123]	*B. sphaericus*	Hydrogelencapsulated spore	Urea, yeast extract, Ca(NO_3_)_2_, 4H_2_O	0.5	−	+
[124]	*B. subtilis*	Steel bar, Hach dr 2400 portable	Urea CaCO_3_ crystals, yeast extracts, NaCl	1.0	+	+
[24]	*B. sphaericus*	Silica gel, polyurethane	Urea, Ca(NO_3_)_2_, 4H_2_O	0.35, 0.25	+	+
[125]	*B. megaterium, B. licheniformi*	Direct with 10^5^ cell/mL of mixing water	Urea-broth culture	0.3	−	+
[126]	*B. sphaericus*	Microcapsule	Urea, calciumnitrate, yeast extract	0.97	−	+
[127]	*B. sphaericus*	Trinocular stereomicroscope	Urea Ca^2+^ ion, CaCl_2_ usage	0.4	+	+
[128]	*S. pasteurii*	Direct with 2–6 × 10^7^ cfu/mL	Mixing water was replaced by urea–yeast extract medium	−	+	−
[129]	*B. sphaericus*	Glass tubes with PU foam	Urea, Ca(NO_3_)_2_	0.3	+	-

## Data Availability

No new data were created or analyzed in this study.
